# Epidemiological, Economic, and Productive Resilience to PRRSV in “Next-Generation” Versus Conventional Intensive Pig Production: A Case Study

**DOI:** 10.3390/ani16193037

**Published:** 2026-09-26

**Authors:** Alice Perrucci, Vanessa Cardana, Alberto Cavagnini, Paolo Capuzzi, Annalisa Scollo

**Affiliations:** 1 Department of Veterinary Sciences, University of Turin, 10095 Grugliasco, Italy; alice.perrucci@unito.it (A.P.); vanessa.cardana@edu.unito.it (V.C.); 2 Struttura s.r.l., 25025 Manerbio, Italy; 3 CP Consulting s.r.l., 25010 Brescia, Italy

**Keywords:** PRRS, virus, biosecurity, performance, indicators, economic impact, sustainability, statistical process control, next-generation

## Abstract

Porcine reproductive and respiratory syndrome virus (PRRSV) remains a constraint to the sustainability of pig production. This 32-month retrospective case study compared the epidemiological, productive, and economic resilience of two intensive farrowing units in Northern Italy, a conventional farm and a Next-Generation Farm (NGF), integrating advanced structural, technological, and management features. Epidemiological resilience was assessed using time-to-stability (TTS), while productive dynamics were monitored through statistical process control, including cumulative sum charts (CUSUMs), for six key performance indicators. Economic indicators were also collected. TTS was 49 weeks in the NGF versus 91 weeks in the conventional farm, prolonged by a second heterologous PRRSV strain. CUSUM monitoring identified early production deviations 9–21 weeks before virological detection, particularly in return-to-service and fertility rates. During TTS, instability in farrowing, return-to-service, and fertility rates were more persistent in the conventional farm (55.2%, 20.7%, and 17.2% out-of-control signals) than in the NGF (0%, 0%, and 6.7%). Post-outbreak, feed, medication, and labor costs per weaned piglet were lower in the NGF than the conventional unit, resulting in lower combined operating costs (€51.30 vs. €57.67/piglet, respectively). CUSUM may complement virological surveillance by providing early evidence of herd-level production deviations preceding PRRSV detection. Overall, the NGF suggested greater resilience through faster epidemiological recovery, greater reproductive stability, and lower operating costs, supporting the potential of NGF systems and a multidimensional approach to resilience and sustainability.

## Simple Summary

This study compared a conventional and a next-generation farrowing system (NGF), equipped with advanced structural, technological, and management features, using a multidimensional approach to resilience. The study evaluated how the two systems responded to naturally occurring porcine reproductive and respiratory virus (PRRSV) outbreaks in terms of disease recovery, production performance, and economic costs, since PRRSV is one of the major challenges affecting the sustainability of pig production. The time to resume producing virus-negative piglets was almost twice as long in the conventional system as in the NGF. Production data were also monitored using statistical control charts to identify changes in six key performance indicators. These analyses revealed early production deviations occurring 9–21 weeks before the virus was detected via laboratory testing, suggesting their potential value for early warning monitoring. Economic results also favored the NGF, which had lower feed, medication, and labor costs per weaned piglet. Overall, the NGF suggested greater resilience across epidemiological, productive, and economic dimensions. Considering these dimensions together may provide a more comprehensive basis for evaluating investments in NGF production systems and their potential contribution to sustainability, particularly in swine-dense areas.

## 1. Introduction

Pig production is one of the world’s major sources of animal protein and is expected to play an increasingly important role in meeting the growing demand for livestock products over the coming decades [1,2]. However, this expansion is occurring in a global context of profound transformation. Beyond improving production efficiency, modern pig farming is increasingly expected to reduce its environmental footprint, improve animal welfare, limit antimicrobial use, and ensure long-term economic sustainability. These evolving expectations have shifted the focus from maximizing productivity alone towards developing production systems capable of maintaining high performance while coping with increasingly complex biological, environmental, and societal challenges [3].

Among these challenges, infectious diseases remain one of the principal constraints to sustainable pig production. In highly specialized and interconnected production systems, pathogen introduction and circulation can rapidly compromise animal health, productive performance and farm profitability, generating substantial biological and economic losses. Consequently, preventing and controlling infectious diseases has become one of the primary objectives of modern swine production.

In this context, biosecurity is widely recognized as the cornerstone of disease prevention. The Food and Agriculture Organization (FAO) identifies biosecurity as a fundamental component of sustainable livestock production, contributing simultaneously to its environmental, economic, and social dimensions [4,5]. Likewise, the European PROHEALTH project highlighted biosecurity, housing design, management practices, and animal welfare among the principal drivers of herd health and productive performance [6].

Nevertheless, even the most advanced biosecurity strategies cannot eliminate the risk of pathogen introduction under commercial conditions. Consequently, the value of biosecurity should not be evaluated solely according to its ability to prevent disease introduction, but also according to its capacity to limit disease circulation and persistence into the farm, preserving system performance when prevention fails. This perspective links biosecurity to production system resilience.

A resilient production system is able to withstand biological disturbances, maintain its essential functions, and rapidly recover following adverse events. Importantly, resilience is reflected not only in animal health, but also in the capacity of a production system to maintain productive efficiency and economic viability during and after disease outbreaks. Therefore, assessing resilience requires the integration of epidemiological, productive, and economic indicators, providing a more comprehensive evaluation of how production systems respond to infectious disease challenges.

Building upon the Next-Generation Biosecurity framework proposed by Dee et al. (2022) [7], we propose the concept of the Next-Generation Farm (NGF) as an evolution of this approach. While the Next-Generation Biosecurity framework primarily focuses on preventing pathogen introduction through advanced biosecurity measures, including air filtration, the NGF extends this concept by integrating optimized building design, precision livestock technologies, welfare-oriented management, and operational strategies into a comprehensive production system. The underlying premise is that the resilience of a production system is not determined by isolated biosecurity interventions alone, but rather by the integration of structural, technological, and managerial components acting synergistically to reduce disease impact and facilitate recovery following infectious challenges.

Among infectious diseases affecting swine, porcine reproductive and respiratory syndrome virus (PRRSV) provides a particularly suitable model for evaluating production system resilience. PRRSV remains one of the most economically important diseases of the global swine industry, causing reproductive failure in breeding herds, respiratory disease in growing pigs, and substantial reductions in productive efficiency [8,9,10]. Owing to its high transmissibility and profound impact on herd performance, PRRSV represents a major biological disturbance to pig production systems, providing an appropriate model to evaluate their ability to withstand and recover from infectious disease challenges.

Although the economic burden of PRRSV and the effectiveness of individual biosecurity measures have been extensively investigated, few studies have compared the epidemiological, productive, and economic response of conventional and next-generation pig production systems under naturally occurring disease conditions. Furthermore, there is a scarcity of field-based studies in Southern Europe, where distinct climatic conditions and environmental stressors may significantly influence disease dynamics and farm economic performance compared to Northern European production contexts. Understanding these differences is essential to determine whether next-generation production systems provide greater resilience than conventional systems, preserving productive efficiency and economic performance even when pathogen introduction cannot be completely prevented.

Therefore, the present study used a naturally occurring PRRSV outbreak as a real-world model to compare the epidemiological, productive and economic dynamics of a next-generation and a conventional pig farm over a 32-month observation period. We hypothesized that the next-generation production system would show greater resilience than the conventional one, resulting in a shorter period of health instability, improved productive performance, and a reduced economic impact following an outbreak.

## 2. Materials and Methods

### 2.1. Systems Description

This retrospective observational case study compared the productive, epidemiological, and economic performance of two commercial farrow-to-wean pig farms representing a conventional and a next-generation production system. Data collection was retrospectively carried out from June 2022 to January 2024 (included), starting from the first weaning batch in June 2022 to the first weaning batch in January 2024. As suggested by Pexas et al. (2020) [11], the selection of the representative farms was based on the availability of high-quality and easily accessible data and on the comparability of their production characteristics. Moreover, both farms belonged to the same owner, thereby minimizing potential confounding associated with differences in management philosophy, investment strategies, and decision-making [12]. To reduce potential sources of confounding, the farms were located in the same geographical area and shared comparable genetics, feeding programs, herd sizes, and stockmanship practices. The primary difference between the two farms was the production system design, with one operating as a conventional intensive farm and the other according to the NGF concept described below.

#### 2.1.1. Study Area and PRRSV Epidemiological Context

A major Italian pig production area was purposefully selected, as a representative setting for the case study. Both systems were located in the province of Brescia, within the Lombardy region, an area in northern Italian highly suited to the heavy pig production for the Protected Designation of Origin ham. The Lombardy region had a census of more than 3.9 million raised pigs at the end of 2023, and it represents the Italian region with the highest pig density (166.5 pigs/km^2^) [13]. In Lombardy, 90.8% of farmed sows are housed in farrowing sites greater than 300 productive sows, out of a total number of 215,000 registered sows. Specifically, the province of Brescia is the second most swine-dense area, with 0.38 pig farms per km^2^ (251.19 pigs/km^2^) [13].

Even if the epidemiological data regarding PRRSV in Italy remain limited [14], the prevalence of infected farms in the Northern part has been estimated at around 90%, although not always associated with clinical signs [15] due to the classification of “stable” for several herds [16,17]. Furthermore, Lombardy represents one of the Italian regions with the highest risk of virus spread, as it is incentivized by a high-density pig population and the agricultural vocation of the area (i.e., high cropland use and low human population and road density) [14].

The next-generation system was located at the geographical coordinates (latitude/longitude) 45°17′12.4″ N/10°11′19.9″ E, with the conventional one at 45°15′20.1″ N/10°13′22.2″ E, i.e., they were located approximately 3 km apart, thereby ensuring exposure to a comparable geographical, climatic, and regional epidemiological context.

#### 2.1.2. Study Farms

The systems under analysis were both indoor, integrated Italian sow farms that were compliant with the minimum welfare standards for the protection of pigs laid down by the Council Directive 2008/120/EC and with the reinforced biosecurity measures listed by the Commission Implementing Regulation (EU) 2023/594 (annex III) on special disease control measures for African swine fever (even if the geographical area was not included in the restricted zones at the time of the study). Both farming systems housed sows of the same genetics and origin (Danish Genetics^®^, Kolding, Denmark), they were owned by the same entrepreneur, and all sows were fed using the same feeding supplier, nutritional formulae, and plan. Farm personnel implemented biosecurity procedures according to standardized protocols and received continuous training from the same veterinarian. Both farrowing sites experienced a PRRSV outbreak during the 32-month observation period, defined if the farm had a PRRSV reverse transcriptase-polymerase chain reaction (RT-PCR)-positive result (see below) [18].

The systems differed primarily in their production system design. The conventional scenario in this study was selected according to published reports describing the average Italian farrowing site belonging to a highly integrated pig production system, typical of Northern Italy [14]. The farm size was 1150 productive sows, in agreement with the most represented size reported by the official Vetinfo report for Lombardy (i.e., greater than 300 sows, see above) and with the average size reported by Drigo et al. (2018) [17] for the same area (1130 productive sows). The farm was built in the 1980s, and its expansion during the last decades, by adding new buildings to older (still functional) ones, led to six heterogeneous barns, as commonly observed in Northern Italy [19,20].

The next-generation scenario was a brand-new and cutting-edge farrowing site that incorporated several sector innovations. The farm was built in 2021 with a single sealed barn, and several recent technologies and innovations to improve different aspects of pig farming were provided: for example, Precision Livestock Farming (PLF) was applied through environmental sensors recording the 24 h/24 temperature, ammonia, and carbon dioxide, with the automated and independent regulation of ventilation for each room. Moreover, the precision feeding was implemented using an electronic feeding station, which dispensed the exact amount of feed to each sow after she was identified by means of a transponder in her ear tag; software for data monitoring and visualization through a real time dashboard was also active. Energy provision was supported by photovoltaic panels (power 52.29 kWp, total area of 283.60) and geothermal temperature management of the inlet air, with the latter allowed to enter the barn (maintained at an internal positive pressure) only after a full filtration with high-efficiency particulate air (HEPA) filters (pre-filter MERV 8, final filter MERV 16) and more than 1000 m of underground channels for geothermal exchanges. Materials entering the facilities were mandatorily subjected to a sanitizing treatment using UV irradiation. Other characteristics regarding welfare were the temporary confinement of farrowing sows and the daily automated straw distribution as environmental enrichment. A complete description of both farms’ characteristics was collected using the governmental Classyfarm checklists on biosecurity (rev. 1/2023; 70 questions) and welfare (rev. 1/2025; 55 questions), summarized and shown in the Supplementary Materials. Below differences regarding both biosecurity and welfare characterizing the conventional and the next-generation scenarios are detailed (Table 1); the definition of “clean” and “dirty” areas for biosecurity was adopted from European Regulation 2023/594.

#### 2.1.3. Health Status and PRRSV Management

At the time of enrollment, both the farms were positive for *Mycoplasma hyopneumoniae* and Porcine circovirus 2 (PCV2) but without evidence of clinical expression. Regarding PRRSV, the conventional scenario was classified as a positive stable with the vaccination herd (category II-vx) [16] at the beginning of the observation period, whereas the Next-Generation Farm was negative, in which replacement gilts were routinely vaccinated before introduction into the breeding herd. The different baseline PRRSV status reflected the distinct health management philosophies underlying the two production systems. While the Next-Generation Farm was designed to maintain a wild-type negative, vaccine-positive breeding herd through strict exclusion of virus introduction, the conventional farm adopted the more common regional approach of maintaining a stable vaccinated herd in which PRRSV circulation was controlled rather than eliminated. Therefore, the initial PRRSV status was considered an intrinsic feature of each production system rather than a source of experimental variability.

In both farming scenarios, breeders’ replacement was conducted by introducing external gilts in an isolation facility inside the farm (quarantine); gilts came from the same PRRSV-negative reproduction genetic center and were aged between 9 and 18 weeks of age. They were moved into the herd just prior to the first insemination, at 33 weeks. At their arrival at the quarantine, a routine PRRSV analysis (serological test on blood samples from 30 animals) [16] was performed to confirm the negative status to PRRSV, and immunization to *M. hyopneumoniae* and PCV2 was applied. After the laboratory confirmation, proper gilts’ immunization against PRRSV was also performed using a modified live vaccine (Reprocyc PRRS EU^®^, Boehringer Ingelheim Animal Health, Ingelheim, Germany). In the conventional farm only, gilts’ acclimatization was performed four weeks later by means of controlled exposure to live herd-derived virus. Prior to introducing the acclimated gilts into the sow herds, PRRSV was investigated again via individual blood sampling and pooled (5 gilts/pool) through a RT-PCR analysis to guarantee that there was no virus shedding when animals were moved to the breeding herd [21].

Regardless of the initial PRRSV status, in both farms, a routinary monitoring PRRSV program was established via the RT-PCR testing of processing fluids for each batch of piglets (majority of litter was included and divided into five pools total: four from piglets born to multiparous sows and one to primiparous sows, protocol modified from Holtkamp et al. (2021) [16]). In the conventional farm and according to the herd monitoring program already in place, blood samples from weaning-age piglets for RT-PCR analysis were also performed by batch (30 piglets; 5 pigs/pool) [16].

#### 2.1.4. PRRSV Outbreak History and Management

During the first two months of 2023, both scenarios experienced a PRRSV outbreak with a field Type 1 (European genotype) strain. Open reading frames (ORF) 5 and 7 genes of both strains were sequenced using procedures detailed by Romeo et al. (2023) [22]. Then, the genetic distance among both viral isolates and the field strain already present in the conventional farm was computed as the pairwise nucleotide difference exclusively between their ORF7 sequences in accordance with regional routine diagnostic protocols, using the Tamura-Nei model [23] (for details see Romeo et al., 2023) [22]. Evolutionary analyses were conducted in MEGA X [24].

In the conventional farm, on 23 January 2023, processing fluids were positive for PRRSV during the routinary RT-PCR analysis. One week later, positive results were also observed for five primiparous sows that showed clinical symptoms possibly referring to PRRSV infection (fever, lethargy, and inappetence) and in aborted fetuses. The genetic distance in ORF7 between the detected PRRSV strain and the local strain was 0.16 and 0.12 when compared to the vaccine strain. As a genetic distance above 0.03 is often considered the cut-off to indicate different origins for the isolates [25], the new strain was classified as heterologous. On 31 August 2023, all the tested processing fluids were RT-PCR-negative again, as well as all piglet blood samples on 19 September 2023. However, intermittent RT-PCR positivity in processing fluids from piglets born to primiparous sows was observed later, until the end of the study.

In the Next-Generation Farm, on 7 February 2023, processing fluids from a batch resulted in positivity for PRRSV after the RT-PCR routinary investigation. During the outbreak, blood samples from sows and external replacement gilts were always negative via RT-PCR. The genetic distance in ORF7 between the detected PRRSV strain and the vaccine strain was 0.14, whereas it was 0.07 (heterologous) when compared to the conventional farm strain. On 7 September 2023, processing fluids became negative again. The following batch showed a positive sample of processing fluids; then, no other positive samples were observed until the end of the study.

Both the farms strictly applied the McRebel program (Management Changes to Reduce Exposure to Bacteria to Eliminate Losses from PRRS; [26]) during the PRRSV outbreak. Briefly, the crossfostering of piglets between litters did not occur after 24 h of age, limiting the movement to piglets in excess in regard to the available teat spaces and strictly within the same room. Moreover, piglets that were unresponsive to an antibiotic treatment were euthanized immediately.

### 2.2. Productive and Economic Outcome Measures

Data on productive performances were collected from the software program PigUp^®^ (Isagri S.r.l., Codogno, Italy), for a total of 9 items: farrowing rate (percentage of females that farrowed from total serviced females), return-to-service rate (percentage of serviced females that returned to estrus), fertility rate (percentage of serviced sows that became pregnant), abortion rate (percentage of pregnant sows that experienced a pregnancy loss during gestation), mummified rate (percentage of mummified fetuses per litter), and pre-weaning mortality (PWM) rate. These items were selected because they represent the key productive indicators (KPIs) most commonly affected during a PRRSV outbreak [10].

Key economic items were obtained from a business intelligence software (Microsoft Power BI, Microsoft Corporation, Redmond, WA, USA) fed by data uploaded and categorized by the same officer, which allowed a comparable collection in both scenarios per trimester. The selection of key parameters was guided by the review published in 2023 and authored by Boeters et al. [27] on cost and revenue components considered in economic analyses of studies on PRRSV: labor costs (obtained from stockmen working hours), breeding costs (sows replacement; insemination costs), feed costs (sows and pre-weaning piglets), medication costs (including treatments to pre-weaning piglets), cull sows’ sales [28,29].

### 2.3. Statistical Analysis

Statistical Process Control (SPC) was used to evaluate temporal changes in productive performance associated with the PRRSV outbreaks, as previously described by Scollo et al. (2022) [30]. XLSTAT software (Addinsoft, New York, NY, USA) was used. Individuals–Moving Range (I-MR) and cumulative sum (CUSUM) control charts were used following Wheeler and Poling’s (1998) [31] and Krieter’s (2009) [32] advice. The herd batch was used as the unit of analysis, in accordance with the 3-week batch production system adopted in both farms. Briefly, the I-MR charts display each single batch measurement over time, whether the CUSUM charts incorporate all information in the sequence of sample values and plots the cumulative sums of the deviations from a target value using samples from all prior observations. Upper control limits (UCLs) and lower control limits (LCLs) were calculated using three standard deviations above and below the center line (the mean of the process values) and, when exceeded, the process was considered out of control (OC). Procedures for setting limits in charts were those suggested by Sanghangthum et al. [33]: the goal is to ensure that the process operates in a state of statistical control, which means that the process is predictable within the limits determined using data from the process. With this purpose, the first 10 batches were used as in-control data points for process analysis, then extending the center line and limits based on these observations to monitor later performance. In both farms, the PRRSV outbreak occurred in batch 14. All eight tests for special cause variation listed below and available in XLSTAT were applied:

Test 1: 1 point beyond 3 standard deviations (out of control signal).

Test 2: 9 successive points on the same side of the centerline (warning signal).

Test 3: 6 successive points increasing or decreasing (warning signal).

Test 4: 14 successive points alternating up and down (warning signal).

Test 5: 2 out of 3 successive points beyond 2 standard deviations (same side, warning signal).

Test 6: 4 out of 5 successive points beyond 1 standard deviation (same side, warning signal).

Test 7: 15 successive points within 1 standard deviation (either side, warning signal).

Test 8: 8 successive points not within 1 standard deviation (either side, warning signal).

The time-to-PRRSV stability (TTS) was defined as the number of consecutive weeks required to produce PRRSV-negative pigs over a 90-day period [34]. This was determined based on RT-PCR failure to detect PRRSV RNA using serum samples from pre-weaning pigs in the conventional farm and processing fluids in the NGF, reflecting the routine diagnostic monitoring protocols established at each site. Key economic items are reported as a descriptive analysis per trimester. Data are shown per sow and per produced piglet. The reference (basic) economic data were acquired six months before the PRRSV outbreak, coincidental to the 10 batches used to create SPC charts limits. For each economic item, the mean of the quarterly values recorded during the post-outbreak period (until the end of the observational period) was calculated separately for the conventional and NGF systems.

Because the objective of the study was to compare the temporal response of two individual commercial production systems rather than estimate population-level effects, no inferential statistical comparisons between farms were performed.

## 3. Results

A total of 86 consecutive sow batches (10,663 productive sows) were monitored over a 32-month period, including the baseline, outbreak, and recovery phases in both production systems.

The NGF recovered PRRSV stability considerably faster than the conventional farm (49 vs. 91 weeks). The prolonged instability observed in the conventional farm coincided with the emergence of a second heterologous PRRSV strain, first detected at batch 31. Over the entire observation period, several productive parameters showed a higher frequency of out-of-control signals, indicating increased process instability. Within the time window defined by the TTS, involving 29 batches in the conventional farm versus 15 in the NFG system, the parameters most frequently exhibiting out-of-control signals were the farrowing rate, abortion rate, mummified rate, and pre-weaning mortality rate. Conversely, the parameters most frequently associated with early production deviation signals (SPC alarm signals occurring before a change in PRRSV status based on RT-PCR testing of processing fluids) [18] were reproductive items, namely the farrowing rate, return-to-service rate, and fertility rate (Figure 1A–F). For early warning parameters, the CUSUM model detected signals associated with PRRSV outbreaks between three and seven batches before the sow farm reported a change in PRRSV status, corresponding to approximately 9–21 weeks. This interval was defined as time-to-detect (TTD) by De Vries and Reneau (2010) [35]. Table 2 summarizes the out-of-control signals and the early deviation rate for each productivity indicator.

Economic key items are expressed in euros (€) per productive sow and per piglet produced, for both the conventional and the NGF. Economic indicators were computed per trimester and expressed as annualized projections, obtained by extrapolating trimester values to a 12-month equivalent. The 2022 annual value was used as the pre-outbreak economic reference for each farm.

When expressed per productive sow, economic indicators did not show a consistent temporal pattern associated with the PRRSV instability windows in either production system (Supplementary Materials, Figure S1). Most cost categories fluctuated independently of the periods of virological instability, with the exception of a marked increase in sow replacement costs in the conventional farm around the detection of the second heterologous PRRSV strain (first quartile 2024). On average, across the entire observation period after the PRRSV outbreak, the NGF was associated with lower average feed (€634.99 vs. €656.56; −3.3%), medication (€129.87 vs. €143.18; −9.3%), and labor (€220.25 vs. €278.35; −20.9%) costs than the conventional system. Average breeding costs were higher in the NGF across both line items, though slightly so for replacement gilt acquisition (€225.00 vs. €218.92; +2.8% in the NGF) and more notably for insemination costs (€42.23 vs. €38.28; +10.3%). Revenue from culled sow sales averaged €72.42 in the NGF and €102.73 in the conventional system (−29.5%). Overall, the combined value of the evaluated economic indicators was 7.9% lower in the NGF than in the conventional system (€1324.75 vs. €1438.02 per productive sow). Compared to the 2022 pre-outbreak baseline, post-PRRSV combined values decreased slightly in the NGF system (€−30.24; −2.28%) but increased in the conventional system (€+72.05; +5.01%).

Economic indicators expressed per weaned piglet showed distinct temporal patterns compared to the analysis per productive sow (Supplementary Materials, Figure S2). These indicators more closely reflected the duration and severity of PRRSV instability. Feed costs exhibited the clearest association with the epidemiological dynamics, increasing during the prolonged instability period and reaching their highest values following the emergence of the second heterologous PRRSV strain. Average feed (€24.65 vs. €25.60; −3.7%), medication (€4.98 vs. €5.62; −11.2%), labor (€8.51 vs. €11.29; −24.7%), and replacement gilt acquisition (€8.76 vs. €9.48; −7.7%) costs were all lower in the NGF, whereas insemination costs were slightly higher (€1.71 vs. €1.52; +12.5%). Revenue from culled sow sales was also lower (€2.70 vs. €4.16; −35.1%). Overall, the combined value of the evaluated economic indicators was 11.0% lower in the NGF than in the conventional system (€51.30 vs. €57.67 per weaned piglet). Compared with the 2022 baseline, post-PRRSV outbreak expenses increased similarly across both systems: by €10.91 (+21.31%) in the NGF system and by €12.51 (+21.14%) in the conventional farm.

## 4. Discussion

Achieving and maintaining a stable productive balance lies at the core of a sustainable swine industry. More recently, this concept has increasingly been framed in terms of resilience, namely the capacity of a production system to withstand disturbances, rapidly recover its functionality, and adapt while maintaining productivity and long-term sustainability. However, this equilibrium is constantly undermined by infectious diseases, which remain a primary bottleneck to sustainable pig production. In this scenario, PRRSV represents a perpetual global threat. By severely compromising herd health, PRRSV impairs both efficiency and economic viability, making this delicate balance exceptionally difficult to reach in high-density swine regions such as Northern Italy [14]. Within the context of this study, resilience was therefore defined as the ability of a production system to withstand a PRRSV challenge, rapidly recover productive stability, and minimize its long-term productive and economic consequences. The Next-Generation Farm (NGF) was conceived to strengthen this resilience through the synergistic integration of structural, technological, and operational measures, representing an evolution of the Next-Generation Biosecurity concept introduced by Dee et al. (2022) [7]. Nevertheless, evidence linking these interventions to farm-level resilience, particularly from an epidemiological, productive, and economic perspective under field conditions, remains limited [12,36,37]. Moreover, the perceived cost of implementing advanced interventions is frequently cited as a major constraint to their adoption, largely due to a lack of comprehensive farm-level economic evaluations demonstrating whether improvements in epidemiological and productive resilience ultimately translate into tangible economic benefits [37,38,39]. This knowledge gap provided the rationale for integrating an economic assessment into the present study alongside the epidemiological and productive evaluations.

Against this background, the present 32-month retrospective case study used a naturally occurring PRRSV outbreak as a real-world model to evaluate the resilience of two commercial farrow-to-wean production systems. A conventional and a NGF farm were compared across three complementary domains: epidemiological resilience, assessed through time-to-stability (TTS); productive resilience, evaluated using Statistical Process Control (SPC); and economic resilience, assessed through post-outbreak production costs.

Before exploring results, the different PRRSV status of the two production systems at the beginning of the observation period warrants specific consideration. Rather than representing a source of bias, this difference reflects the distinct production philosophies of the two systems. The conventional farm followed the common endemic management strategy adopted in many European breeding herds, where PRRSV is managed to maintain herd stability. Conversely, the NGF was designed according to a disease-exclusion strategy, aiming to prevent field-virus introduction while maintaining immunity through gilt vaccination only. Consequently, the comparison performed in the present study should not be interpreted as a comparison between two farms differing only in PRRSV status, but rather between two production systems based on different PRRSV management strategies. The observed differences in epidemiological resilience are therefore best interpreted within this broader production-system context rather than as the consequence of PRRSV status alone.

The first resilience indicator evaluated was time-to-stability (TTS), a widely recognized metric of herd recovery following a PRRSV outbreak because it reflects the duration of viral circulation within the breeding herd and the effectiveness of control measures. In this study, the NGF re-achieved stability in 49 weeks, compared to 91 weeks for the conventional farm, where the instability period was prolonged by the introduction of a second heterologous PRRSV strain during the observation period. Consistent with these findings, TTS reported by other authors in previous field studies varies considerably according to the control strategy adopted, monitoring protocol, and epidemiological scenario, with values ranging from 18 to 163 weeks [34,40,41].

The 42-week difference observed in this study, with the conventional farm taking nearly double the time to recover compared to the NGF, may suggest a difference in epidemiological resilience following a natural PRRSV challenge, prompting consideration of the structural and management factors that may have contributed to the shorter TTS in the NGF. However, these findings must be interpreted with caution. In a two-farm case study, system-level effects cannot be fully disentangled from farm-specific PRRSV baseline conditions, such as the conventional farm being PRRSV-positive and stable at baseline versus the NGF being wild-type negative and vaccine positive, as well as the occurrence of a second heterologous PRRSV introduction in the conventional herd during the observation period. While these baseline and epidemiological differences reflect real-world production dynamics, they may have independently influenced outbreak severity and recovery timelines. Furthermore, while the contribution of individual structural or managerial features cannot be isolated in a field case study, one plausible contributing factor for this difference lies in the integrated design of the NGF, particularly its internal air management strategy. Unlike the conventional system, the NGF combined high-efficiency air filtration with room-specific ventilation and independent airflow, features that may have limited within-farm viral dissemination once PRRSV had entered the herd. The protective effect of mechanical air filtration against PRRSV introduction has been extensively documented [42,43,44,45], with risk reductions of up to 20-fold [46] and decreases in barn virus concentrations ranging from 57% to 80% [45]. However, once a pathogen has entered a production site, limiting its circulation within the farm remains equally critical [47]. The role of airflow in the within-farm transmission of several pathogens, including PRRSV, is well-documented [47,48,49,50,51,52], with indoor bioaerosol dynamics contributing to non-contact transmission between animal groups [53]. Accordingly, optimized ventilation strategies combining individual room ventilation with compartmentalized airflow can limit air exchange between infected and susceptible sections [53,54], with reductions in disease transmission of up to 43.2% compared with standard ventilation configurations reported by Safari et al. (2024) [52]. This structural feature adopted in the NGF may therefore have contributed to creating controlled microenvironments reducing both intra-farm PRRSV circulation and the risk of new strain introduction, possibly contributing to its shorter TTS. Although the contribution of ventilation cannot be isolated from the other structural and management features of the system, its potential role in strengthening epidemiological resilience may be particularly relevant in high-density swine production areas, where continuous infection pressure increases the risk of pathogen introduction and subsequent within-farm spread [14].

Despite the advanced disease-exclusion measures implemented in the NGF, field PRRSV introduction still occurred. Although the specific source and route of introduction could not be retrospectively identified in either farm, an unidentified human-related breach in the application of biosecurity procedures was considered the most plausible explanation. Indeed, even highly controlled production systems remain dependent on the correct and consistent implementation of biosecurity protocols by personnel and other individuals interacting with the farm. Nevertheless, alternative routes of introduction cannot be completely excluded. This observation highlights that structural and technological barriers can reduce, but cannot entirely eliminate, the risk of pathogen introduction [42,43,44,45].

To investigate whether the differences in epidemiological resilience were also reflected in production dynamics, key production indicators (KPIs) were analyzed using Statistical Process Control (SPC), a quality control methodology designed to monitor performance over time. SPC is especially suited to production systems where data are routinely recorded as continuous time series, thereby enabling evaluations of process stability [55]. Within this framework, SPC facilitates the real-time monitoring of production metrics and prompt detection of deviations from baseline performance, a critical capability for systems where long-term operational stability is paramount for resilience [56], such as sow herds.

For the purpose of discussing their temporal behavior, KPIs here are grouped into pre-farrowing indicators (farrowing rate, return-to-service rate, and fertility rate) and post-farrowing indicators (abortion rate, mummified fetus rate, and PWM). The first relevant finding was the different behavior of pre- and post-farrowing indicators during the period preceding virological confirmation of PRRSV circulation. Pre-farrowing reproductive parameters triggered out-of-control (OC) signals (indicating a loss of productive stability) prior to PRRSV detection in processing fluids, in particular in the NGF system. Specifically, OC signals were detected before the outbreak for the farrowing rate (7.7% in conventional vs. 15.4% in NGF), return-to-service rate (7.7% vs. 38.5%), and fertility rate (7.7% vs. 38.5%). This anticipatory pattern was further supported by the CUSUM charts, which generated consistent early-warning indicators across all three parameters in both production systems: signals occurred 3–7 batches before the change in PRRSV status, corresponding to approximately 9–21 weeks. Early alarms in the return-to-service rate prior to a PRRSV outbreak align with observations by Engler et al. [57], who similarly reported early shifts in this parameter up to 3.5 months before PRRSV detection in two commercial herds, raising the possibility that reproductive performance monitoring could be further evaluated as an early indicator of disturbances in herd health.

During the TTS window, pre-farrowing KPIs continued to display process instability through OC signals, although with markedly different frequencies between the two production systems. Notably, the conventional farm exhibited a higher overall alarm frequency compared to the NGF (+55.2% for farrowing rate, +20.7% for return-to-service rate, and +10.5% for fertility rate). Thus, both systems experienced a loss of productive stability, but with different temporal patterns: in the NGF, pre-farrowing reproductive instability emerged predominantly before virological confirmation, whereas in the conventional farm, it became markedly more evident during the TTS window. Changes in farrowing and return-to-service rates associated with PRRSV challenge have also been reported by other authors under different epidemiological and biosecurity conditions [58,59].

Contrary to what was observed for pre-farrowing KPIs, post-farrowing items (abortion rate, mummified fetus rate, and PWM) showed little evidence of process instability before PRRSV detection. This stands in contrast to the findings of Pedro Mil-Homens et al. (2024) [18], who reported that the weekly PWM, followed by abortion rates, generated an early CUSUM signal before PRRSV confirmation in 71% and 64% of the evaluated outbreaks, respectively, with signals occurring up to 8 weeks before detection in individual outbreaks. Similarly, Silva et al. [60] observed that the early deviation rate was highest for abortions (90%), identifying outbreaks up to 4 weeks prior to clinical onset. Conversely, during the PRRSV outbreak in the present study, all post-farrowing KPIs did display OC signals in both production systems. This is consistent with the well-documented impact of PRRSV on post-farrowing performance [28,58,61].

Taken together, these findings support the hypothesis that selected production KPIs may provide consistent early-warning information before the virological confirmation of PRRSV circulation. In the present study, this was particularly evident for return-to-service and fertility rates in the NGF, consistent with previous reports identifying reproductive performance changes before PRRSV detection. These findings warrant further investigation in larger studies to determine whether such early deviations can reliably serve as field signals prompting targeted diagnostic testing and, ultimately, the earlier implementation of appropriate bio-containment measures. Conversely, the marked variability observed for the other KPIs currently limits their reliability as consistent early-warning indicators. Nevertheless, such performance shifts must be interpreted with caution, as they are not PRRSV-specific. Confounding management factors, including improper insemination timing, suboptimal mating practices, or variable semen quality, could also influence these metrics.

The greater detectability of pre-detection deviations in the NGF may partly reflect the characteristics of SPC itself: when baseline performance is highly stable, even relatively small departures from expected performance can become more readily detectable. In the present study, this may have enhanced the visibility of early productive disturbances in the wild-type negative, vaccine-positive NGF. Importantly, when the pre- and post-detection periods are considered together, the overall duration of productive instability was shorter in the NGF, consistent with its faster epidemiological recovery. This contrasting trajectory suggests that productive resilience may be reflected not by the absence of instability, but by the capacity of the system to contain its duration and restore productive stability more rapidly.

In this context, SPC provides a useful framework for characterizing productive resilience, as it describes not only when a production process departs from its expected state, but also how persistent that deviation is and when stability is restored. Overall, applying SPC to livestock time series provides actionable, fact-based information for routine herd management, complementing continuous laboratory diagnostics [18,60]. Under real-world conditions, implementing an SPC framework in NGF systems can be conceptualized as a three-step operational sequence: (1) early signal detection, (2) targeted investigation of root causes, and (3) timely management or biosecurity intervention.

1. Signal detection. Control charts allow for the routine monitoring of multiple key production parameters rather than relying on a single indicator, as tracking concurrent variables may enhance early detection capacity. In this context, the value of an early-warning system must be balanced against the possibility of false alarms. Krieter et al. [32] showed that increasing the sensitivity of CUSUM to detect small changes in sow-herd performance generally shortened the time to signal at the expense of a higher false-positive rate. However, they also emphasized that, in farm monitoring, rapid identification of a true process shift may be more relevant than completely avoiding false-positive signals, because an alarm prompts investigation without interrupting the production process.
2. Investigation of underlying causes. SPC alarms are not cause-specific, serving as a trigger for an in-depth investigation to identify causes possibly stemming from both infectious pathogens and non-infectious managerial variations [35]. For instance, upon receiving an early warning signal from pre-farrowing reproductive parameters, targeted laboratory testing can be immediately deployed to rule out major swine reproductive pathogens, alongside feed analyses and audits of recent management or environmental changes. When PRRSV is suspected, the diagnostic investigation may be expanded to appropriate animal groups and sample types to assess whether viral circulation is already occurring, rather than relying exclusively on routine processing-fluid surveillance.
3. Timely interventions. Following SPC alarms, timely interventions can be implemented immediately, including the reinforcement of existing internal biosecurity protocols and management practices [30]. In particular, herd managers should review animal, personnel, and equipment movements between production areas, reinforce McRebel management guidelines [26], and minimize iatrogenic transmission by changing needles between individual animals during injectable treatments and vaccination [61]. If PRRSV circulation is confirmed, the herd veterinarian may evaluate a coordinated stabilization strategy tailored to the farm’s epidemiological status. This might include temporary herd closure to the introduction of replacement animals, strict control of animal and personnel flows between production areas, and whole-herd vaccination where appropriate [62]. In the present study, SPC signals preceded virological confirmation by 9–21 weeks, suggesting that routine performance monitoring may provide an opportunity to initiate diagnostic investigations and precautionary control measures before infection is identified through conventional surveillance.

In this way, SPC provides a practical link between routinely collected production data and proactive herd-health management, supporting the capacity of production systems to detect, respond to, and recover from disturbances.

The performance instabilities observed in this study are expected to have financial implications. This is supported by previous studies estimating that losses occurring at the sow-herd level account for 54.5–60.9% of the total economic burden associated with PRRSV [8,9,29]. Combining the observed productive shifts with financial indicators is therefore essential to evaluate the real-world resilience of these production systems. However, it is important to highlight that economic assessment reflects the different management and philosophies of both farming systems, rather than the isolated economic impact of PRRSV.

Findings from this study suggest that the perceived magnitude of PRRSV-induced financial disruptions is influenced by the unit of expression used. Expressing economic indicators per productive sow primarily reflects fixed herd overheads, which remain relatively stable regardless of sanitary shifts, thereby diluting the perceived clinical impact of the disease. Indeed, when calculated per productive sow, most economic items showed no consistent temporal relationship with the PRRSV instability windows. This is biologically and economically plausible because many sow-level expenditures are primarily related to maintaining the breeding herd and therefore remain relatively insensitive to short-term reductions in piglet output. Conversely, costs expressed per weaned piglet more closely reflected the epidemiological dynamics. Expressing costs per piglet incorporates productive output into the denominator; consequently, when fewer piglets are produced, operating expenditures are distributed across fewer units, increasing the cost per weaned piglet. This denominator effect is consistent with previous economic assessments identifying reduced piglet output as a major contributor to PRRSV-associated losses [8,27,28,29]. Within this framework, feed costs per weaned piglet showed the clearest temporal correspondence with PRRSV instability, peaking after the introduction of the heterologous strains and closely mirroring the duration of the instability period. This pattern is consistent with feed representing a major component of pig-production costs and illustrates how impaired reproductive output can increase feed expenditure when expressed per unit of output [27]. Medication and labor costs were also lower on average in the NGF during the post-outbreak period, although their temporal patterns were less closely aligned with the virological instability windows.

The comparison with the 2022 pre-outbreak reference further highlights the importance of the unit used to express economic changes, with per-piglet values providing a more realistic representation of the economic consequences associated with reduced productive output. Post-outbreak per-piglet metrics indicated an expense increase of approximately 21% in both systems (+21.31% in the NGF, and +21.14% in the conventional farm). Conversely, per-sow evaluations yielded apparently modest variations (−2.28% and +5.01%, respectively). In this case, the slight reduction in post-outbreak costs per sow observed in the NGF should be contextualized against the 2022 reference, which corresponded to an early operational phase of the farm following its establishment in 2021. Therefore, part of the subsequent economic variation may reflect the progressive stabilization of the production system rather than the effect of PRRSV alone. Importantly, the similar relative increases observed per weaned piglet should not be interpreted as evidence of an equivalent economic response to PRRSV, as these changes occurred from different baseline cost levels and within different productive and epidemiological trajectories. By the same rationale, the observed variation in breeding costs may also be interpreted in this context.

Overall, across the entire post-outbreak period, the NGF showed a more favorable economic profile for several major operating items. Compared with the conventional farm, average feed, medication, and labor costs were 3.7%, 11.2%, and 24.7% lower per weaned piglet, respectively; replacement gilt acquisition was also 7.7% lower per piglet. Only insemination costs were 12.5% higher, with a reduced income from culled revenue. Overall, these differences resulted in a lower average combined operating cost for each weaned piglet in the NGF (€51.30/piglet) than in the conventional farm (€57.67/piglet). This more favorable cost profile may be interpreted within the broader production philosophy of the NGF, which is also based on a disease-exclusion strategy implemented through stricter biosecurity protocols aimed at maintaining a high sanitary status and limiting the productive consequences of disease challenges. Although the contribution of individual measures cannot be disentangled in the present study, previous economic assessments support the potential value of disease-prevention and biosecurity strategies in reducing disease-associated production losses and costs [27,29].

A primary strength of this study lies in the use of real-world field data obtained directly from commercial settings, rather than relying on theoretical assumptions from the literature or expert opinion. To our knowledge, this is the first study from a Southern European context, specifically the Italian pig production sector, to simultaneously integrate epidemiological, productive, and economic outcomes following a natural PRRSV challenge, illustrating the value of evaluating resilience as a multidimensional property across distinct production systems. A further strength is the inclusion of real-world economic data from a production system in which advanced disease-prevention and biosecurity measures are integrated into routine commercial operation. This is particularly relevant because the economic performance of such interventions remains poorly documented under field conditions and their perceived costs may represent a barrier to wider adoption. The more favorable post-outbreak operating-cost profile observed in the NGF therefore provides field-based evidence consistent with previous studies suggesting potential economic benefits of investments in biosecurity and disease prevention [27,29], although the present study was not designed to establish a formal return on investment.

At the same time, the findings should be interpreted within the constraints inherent to a retrospective two-farm case study, as the small sample size inherently limits the generalizability of the results to the broader swine population. Although the farms were purposefully selected to minimize potential confounding related to production and management characteristics, they represented distinct production systems rather than experimentally standardized units, and the influence of unmeasured farm-specific factors cannot be excluded. Therefore, the observed differences should be interpreted as field-based evidence from the two production systems investigated rather than as population-level estimates of their comparative resilience. Moreover, the PRRSV strains involved were not identical; differences in strain characteristics may influence the magnitude of production losses [28,63], and no data regarding co-infections, notably associated with PRRSV infection [10,38], were available.

Future research should therefore extend this framework to a larger cohort of NGF, incorporating epidemiologic, productive, and economic parameters. Formal cost–benefit analyses incorporating capital and operating expenditures would be required to establish whether the observed operational advantages offset the investments associated with NGF implementation. Additionally, evaluating non-monetary externalities, such as potential environmental, social, and animal welfare co-benefits, would provide a more comprehensive cost–benefit assessment. This broader framework would help quantify the full value of NGF investments beyond strictly internal financial returns.

## 5. Conclusions

This case study provides field-based evidence that resilience to PRRSV should be evaluated as a multidimensional property encompassing epidemiological recovery, productive stability, and economic performance. Under the conditions investigated, the NFG system achieved shorter time-to-stability and showed lower persistence of instability in some KPIs following PRRSV detection than the conventional system. The integration of SPC for longitudinal data monitoring proved to be a valuable tool for early production deviation and herd management, supporting the transition toward more sustainable swine production. The economic analysis further showed that PRRSV-related production disturbances were more clearly reflected when costs were expressed per weaned piglet rather than per productive sow. Within this output-based framework, the NGF maintained a more favorable post-outbreak operating-cost profile, particularly for feed, medication, and labor. Taken together, these findings provide evidence-based insights to support informed decision-making and support the potential of investments in next-generation infrastructures, particularly in high-density swine production areas. Ultimately, this next-generation framework represents a promising path toward developing more resilient and sustainable swine production systems.

## Figures and Tables

**Figure 1:**
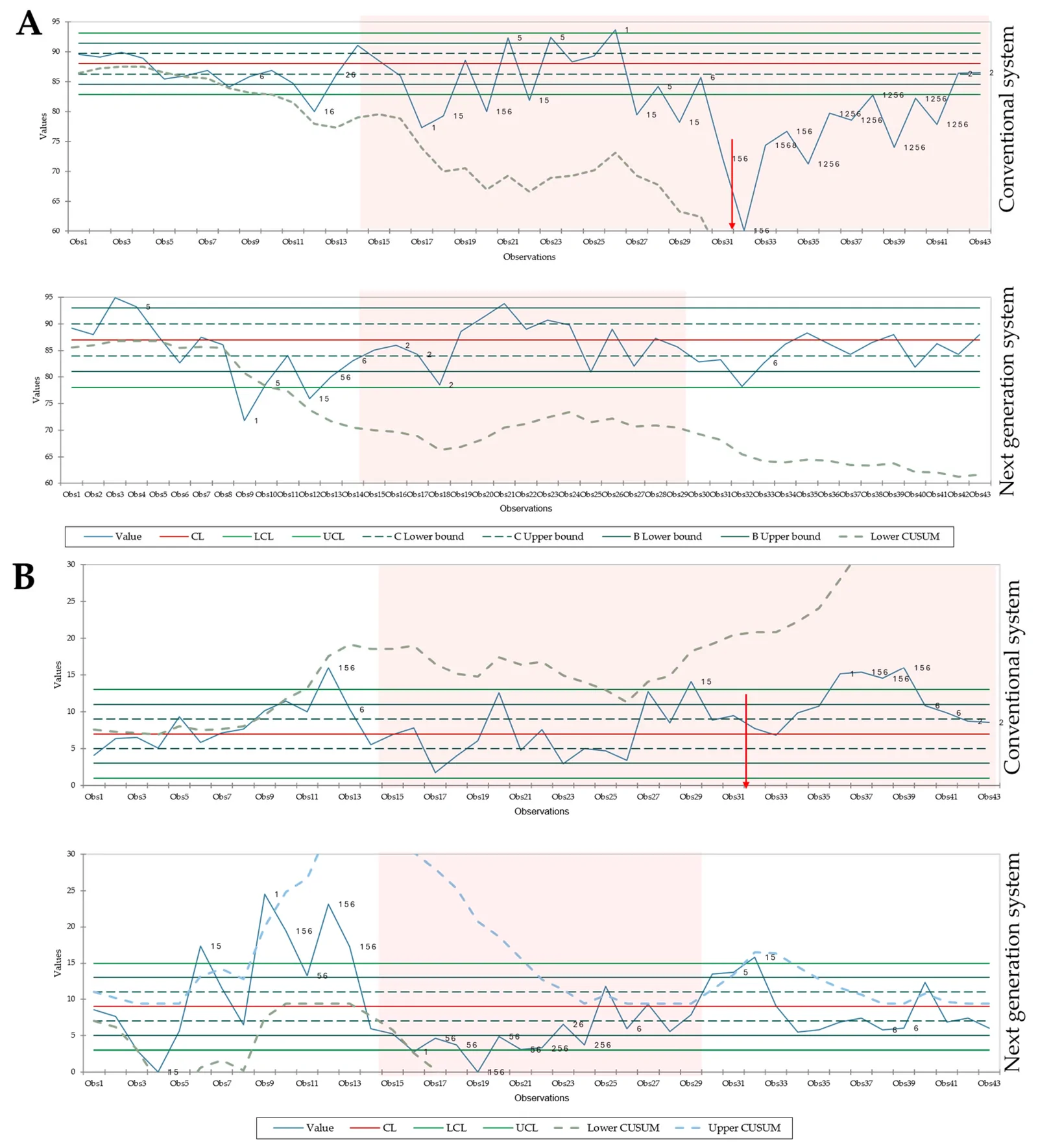

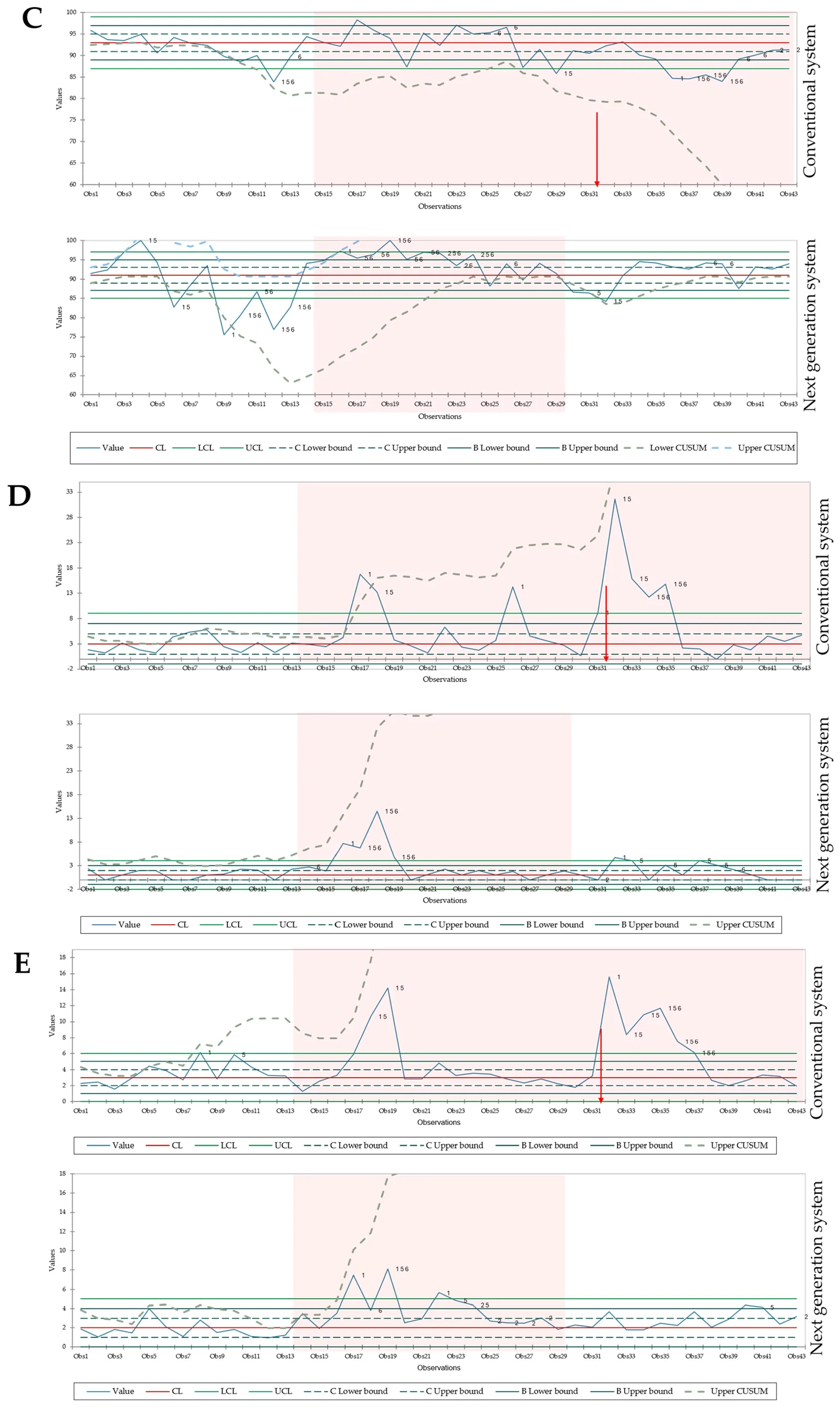

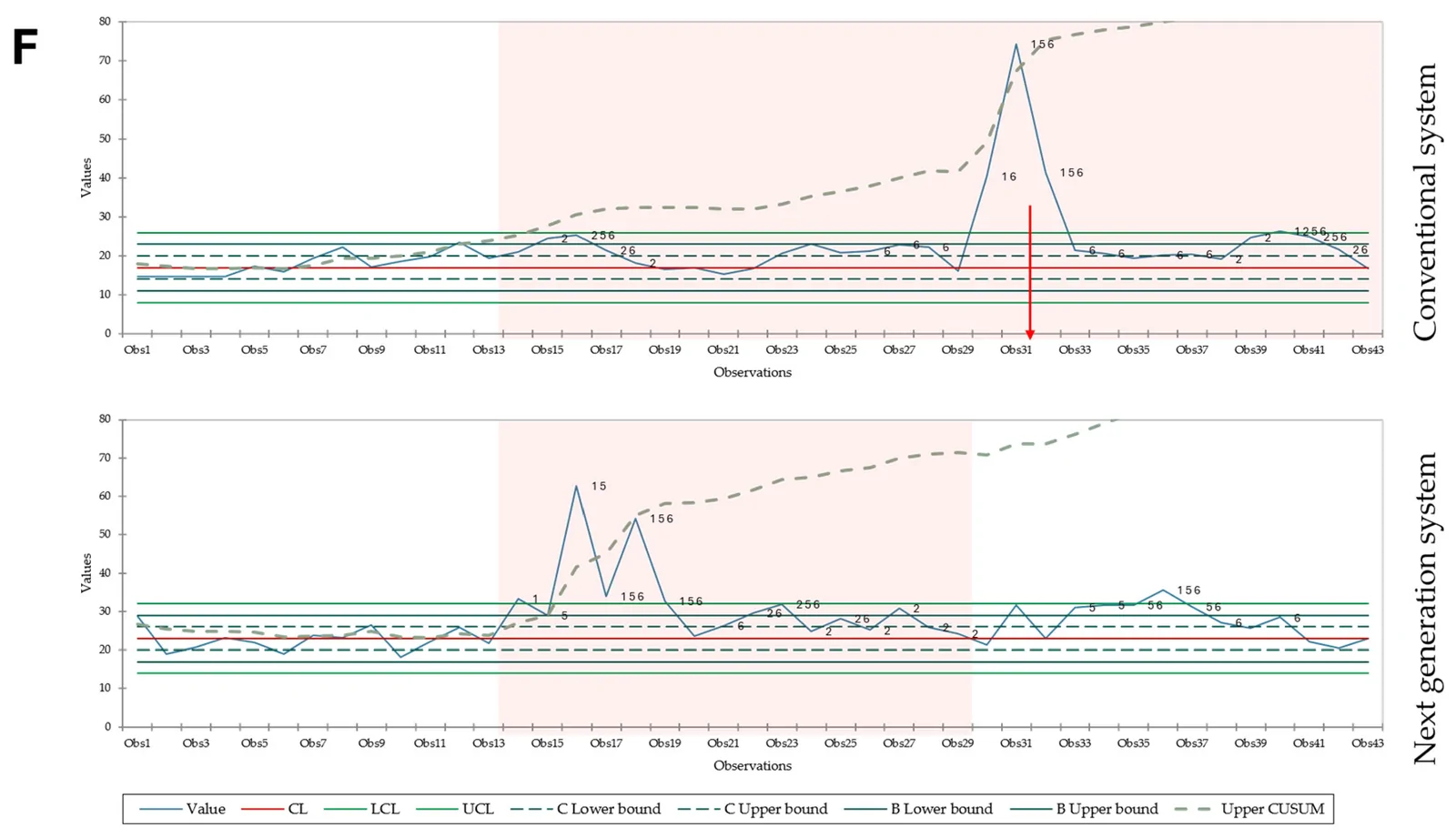
Individuals–Moving Range (I-MR) and cumulative sum (CUSUM) control charts for (**A**) farrowing rate (%), (**B**) return-to-service rate (%), (**C**) fertility rate (%), (**D**) abortion rate (%), (**E**) mummified rate (%), and (**F**) pre-weaning mortality rate (%) recorded at batch level in the conventional (**upper panel**) and the Next-Generation (**lower panel**) Farm. Solid blue lines represent individual batch values, while dashed lines represent the corresponding CUSUM statistics. The central red line indicates the process mean, calculated using the first 10 batches, and upper and lower control limits (green lines) were set at ±3 standard deviations from the mean, while the dashed blue lines indicate ±1 standard deviation from the mean. The light red shaded area indicates the period from the first detection of a heterologous PRRSV strain at farm level to the achievement of stability, defined as the production of PRRSV-negative piglets over a 90-day period. The red arrow indicates the occurrence of a second PRRSV outbreak in the conventional farm. Numbers displayed on the individual charts correspond to the specific chart alarm signals (see the text above).

**Table 1:** Functional comparison between the conventional and the Next-Generation Farm (NGF) production systems.

Functional Domain	Specific Feature	Conventional Farm	Next-Generation Farm (NGF)	Checklist Reference *
External biosecurity	Risk of contact between pigs, feeding and bedding with other animals	The entire farm perimeter was surrounded by a stock-proof fence; nevertheless, small wild animals (e.g., rodents, birds, and insects) could enter the facilities and access pigs and straw. Feed was stored in closed, animal-proof silos.	The farm consisted of a single completely sealed building including the material storage area; no animal species could come into contact with pigs, feed, or bedding. Full HEPA air filtration further reduced the risk of airborne introduction of insects and other biological contaminants.	Biosecurity n. 9, 10, 42, 51
	Vehicle access and loading/unloading procedures	No vehicles were allowed inside the farm perimeter except trucks for live-animal transport. After passing through a disinfection gate, vehicles followed a dedicated fenced route separated from the clean area. Mobile loading ramp.	No vehicles entered the clean area. A permanent loading/unloading bay separated the clean and dirty zones, eliminating the need for vehicle disinfection gates. Permanent concrete loading ramp.	Biosecurity n. 28, 32
Internal biosecurity and compartmentalization	Movement of people and animals	People and pigs moved outdoors between separate barns while remaining within the clean area. The same footwear was used across buildings (except quarantine), with no intermediate disinfection points or mandatory hand washing/disinfection before entering each building.	Single enclosed building with no outdoor movements between production areas. Internal circulation occurred entirely within the facility. Dedicated footwear for quarantine.	Biosecurity n. 14, 15
	Cleaning and disinfection of facilities	Buildings of different ages with heterogeneous layouts; several worn surfaces reduced the effectiveness of cleaning and disinfection procedures.	Smooth, continuous, and washable surfaces specifically designed to facilitate effective cleaning and disinfection.	Biosecurity n. 11
	Manure management	Interconnected manure pits among all barns (including quarantine). Slurry from the farm together with approximately 30% slurry from external farms was injected into surrounding agricultural land.	Independent manure pits for each room. Only slurry produced within the farm was distributed through an underground pipeline system	Biosecurity n. 49, 57, 60
Air management and environmental protection	Ventilation and air handling	Conventional forced ventilation with seasonal cooling for lactating sows. Alarm system activated in case of failure.	Positive-pressure ventilation with complete HEPA filtration (pre-filter MERV 8; final filter MERV 16), independent air management for each room, and more than 1000 m of underground geothermal air-exchange channels. Alarm system activated in case of failure.	Biosecurity n. 9, 57; Welfare n. 27, 41
Precision Livestock Farming (PLF)	Environmental monitoring and automated control	Conventional environmental monitoring and manual adjustment of housing conditions.	Continuous 24 h monitoring of temperature, ammonia, and carbon dioxide, with automated room-specific ventilation control and real-time environmental monitoring.	-
	Precision feeding and digital management	Conventional feeding according to the herd feeding program.	Electronic individual feeding through ear-tag transponder identification, automatic feed allocation, and real-time production dashboard for data visualization and decision support.	-
Animal welfare	Tail docking	Tail docking was routinely performed in approximately 80% of piglets in accordance with Council Directive 2008/120/EC.	Tail docking was never performed.	Welfare n. 14
	Gestation and farrowing facilities	Housing complied with Council Directive 2008/120/EC. Conventional farrowing crates with fully slated flooring.	Temporary confinement only (4 days after insemination and 7 days after farrowing). Gestating sows had 2.5 m^2^ per animal. Farrowing pens measured 7.2 m^2^, and approximately 70% of the floor consisted of solid flooring.	Welfare n. 35, 36, 37, 51
	Environmental enrichment	Wooden block and metal chain are available in all pens. Paper scraps provided as nesting material.	Daily provision of short, chopped straw as nesting material and environmental enrichment for all sow categories.	Welfare n. 49, 52
Environmental sustainability	Energy management	Conventional electricity supply.	Renewable energy supplied by a 52.29 kWp photovoltaic system (283.6 m^2^).	-
	Climate sustainability	Conventional air conditioning based on mechanical ventilation.	Geothermal air conditioning through more than 1000 m of underground heat-exchange channels integrated into the ventilation system.	-

* See the complete checklists in the Supplementary Materials.

**Table 2:** Summary of out-of-control signals and early deviation signals identified via SPC analysis for each productive parameter in the conventional and Next-Generation Farms. Out-of-control signals are reported as the number (and percentage) of batches showing SPC alarms before the PRRSV outbreak and during the time-to-stability (TTS) window. Early deviation is expressed as the number of batches preceding the PRRSV outbreak in which a signal was detected by the CUSUM model, before changes in PRRSV status based on processing fluids RT-PCR testing.

	Out-of-Control Signals Before the PRRSV Outbreak		Out-of-Control Signals During the Time-to-Stability Window		Batches Before the PRRSV Outbreak Showing an Early Deviation Signal in the CUSUM Model	
Items	Conventional Farm	NGF	Conventional Farm	NGF	Conventional Farm	NGF
Farrowing rate	1/13 (7.7%)	2/13 (15.4%)	16/29 (55.2%)	0/15 (0.0%)	5	5
Return to service rate	1/13 (7.7%)	5/13 (38.5%)	6/29 (20.7%)	0/15 (0.0%)	3	7
Fertility rate	1/13 (7.7%)	5/13 (38.5%)	5/29 (17.2%)	1/15 (6.7%)	3	7
Abortion rate	0/13 (0.0%)	0/13 (0.0%)	7/29 (24.1%)	4/15 (26.7%)	0	0
Mummified rate	1/13 (7.7%)	0/13 (0.0%)	8/29 (27.6%)	3/15 (20.0%)	6	0
Pre-weaning mortality rate	0/13 (0.0%)	0/13 (0.0%)	3/29 (10.3%)	5/15 (33.3%)	0	0

## Data Availability

The data supporting the findings of this study are available from the corresponding author upon reasonable request. The data are not publicly available due to their commercially sensitive nature.

## Supplementary Materials

The following supporting information can be downloaded at: https://www.mdpi.com/article/10.3390/ani16193037/s1.

## Abbreviations

The following abbreviations are used in this manuscript:

NGF	Next-Generation Farm
PRRSV	Porcine reproductive and respiratory virus
RT-PCR	Reverse transcriptase-polymerase chain reaction
PCV2	Porcine circovirus 2
ORF	Open reading frame
PWM	Pre-weaning mortality
KPIs	Key productive indicators
SPC	Statistical Process Control
I-MR	Individuals–Moving Range
CUSUM	Cumulative sum control
UCL	Upper control limit
LCL	Lower control limit
OC	Out of control
TTS	Time-to-stability
TTD	Time-to-detection
