# Supplementary material for: Epidemiological, Economic, and Productive Resilience to PRRSV in “Next-Generation” Versus Conventional Intensive Pig Production: A Case Study
Source: Animals (Basel). 2026 Sep 26;16(19):3037. doi: 10.3390/ani16193037 (PMC13663572; doi:10.3390/ani16193037)

Supplementary Materials

Table S1. Classyfarm checklist Biosecurity Assessment: high-capacity Indoor Pig Farms (REV. 1/2023).

Biosecurity checklist: general section

		Conventional farm	Next-generation farm
1	The company has a dedicated area, placed before the entry barrier for the parking of vehicles of farm staff and/or visitors?	Yes, the farm was provided with a parking area situated before the entry barrier.	Since the farm was a completely sealed barn, the dirty area coincided with the area outside the farm walls, explaining why there was no need for an entry barrier.
2	At the entrance are present and clearly visible no trespassing signs for non-authorized people?	At the entrance no-trespassing signs were well visible.	On the only access point no-trespassing signs were well visible.
3	The company has gates or bars suitable for avoiding direct and uncontrolled entry of vehicles and/or people?	An automatic and electrical gate constituted the entry to the farm.	At the only access point a security PIN had to be entered to open the door.
4	The farm has a filter area, with mandatory access for farm personnel and visitors?	Yes, there was a filter area as the only access point for visitors and personnel.	Yes, there was a filter area as the only access point for visitors and personnel.
5	In the filter zone, is there a clear separation between the dirty area and clean area?	Yes, in the filter area there was a very clear separation between the dirty area and clean area.	Yes, in the filter area there was a very clear separation between the dirty area and clean area.
6	Staff/visitors wear clothing or overalls and disposable footwear that is used exclusively in the farm?	Clothes and footwear dedicated uniquely to the clean area were available for far personnel and visitors.	Clothes and footwear dedicated uniquely to the clean area were available for farm personnel and visitors.
7	Is it forbidden for the staff/visitors to bring food inside the farm for personal use?	No, except for animal protein origin food. The staff/visitors are allowed to eat only in the canteen of the farm.	No, except for animal protein origin food. The staff/visitors are allowed to eat only in a specific area of the farm.
8	Is there a plan, with barns and pens uniquely numbered, by which it is	Yes, at the office the documents are available.	Yes, at the office the documents are available.

	possible to verify the unidirectional animal flows in the farm and to identify the animal groups?		Moreover, a map hanging on the wall was available for staff/visitors.
9	Premises and buildings of establishments are so constructed that no other animal can come in contact with reared pigs or their feed and bedding material?	All the farm area was surrounded by a livestock-fence area; nevertheless, rats and pigeons could enter the facilities.	Since the barn was completely sealed, no other animal species could come in contact with reared pigs, and stocking areas were provided with livestock-proof fences.
10	Premises where pigs are kept and buildings where feed and litter are stored are delimited by a livestock-proof fence?	Yes, a livestock-proof fence delimited all the farm area.	There was a livestock-proof fence delimiting the feed stocking areas. All other materials that need to enter the facility, including litter, must undergo UV irradiation.
11	Do the housing facilities have walls, floors, and fixtures that are sealed and in good condition, without any gaps, and that can be effectively cleaned and disinfected?	As the housing facilities were old, they were not all in good condition and several gaps were present, making more difficult an effective cleaning and disinfection of the structures.	The housing facility was completely sealed, in good condition, and provided with smooth and continuous surfaces that could be effectively cleaned and disinfected.
12	Is there a cleaning and disinfection procedure for the housing facilities, environments, and equipment, and if necessary, at the end of each production cycle (even for specific sectors)?	Yes, there was a cleaning and disinfection procedure for housing facilities, environments and equipment, always realized at the end of each production cycle.	Yes, there was a cleaning and disinfection procedure for housing facilities, environments and equipment, always realized at the end of each production cycle.
13	Is the area surrounding the barns clean, covered with gravel or mowed grass, and free of obstructions, objects, equipment, machinery, vehicles, etc., that are unrelated to the functionality and management of the farm?	The area surrounding the barns was totally paved and free of useless objects.	The area surrounding the barn was clean and covered with gravel. No object or machinery was present.
14	Does the farm have points for changing or disinfecting footwear between the different barns?	No, there was a unique point for disinfecting footwear for all barns, except for the	Since the farm consisted of a single sealed barn, no footwear changing or disinfection was necessary;

		quarantine barn, where there was dedicated footwear.	except for the quarantine room, where dedicated footwear was provided for staff/visitors.
15	Does the farm have points for disinfecting and washing hands between the different barns?	No, there were not any disinfecting and washing hands between the different barns	No disinfecting and washing hands points were required since there was a single barn.
16	Is a deratization plan planned and documented?	Yes	Yes
17	Is deratization carried out by an external specialized company?	Yes, it is carried out by an external specialized company.	Yes, it is carried out by an external specialized company.
18	Is a pest control plan in place and documented?	Yes	Yes
19	Is pest control carried out by an external specialized company?	Yes	Yes
20	Is there documentation related to external or internal training courses on biosecurity and the risks of introducing infectious and transmissible diseases in animals that are subject to reporting?	Certificates from attended training by personnel were available.	Certificates from attended training by personnel were available.
21	Is there a documented vaccination prophylaxis plan?	Yes, there was a documented vaccination prophylaxis plan updated, if necessary, by the veterinarian.	Yes, there was a documented vaccination prophylaxis plan updated, if necessary, by the veterinarian.
22	Is there a hygienic and sanitary practice for managing equipment used for vaccination prophylaxis and individual or group therapeutic treatments?	Yes, there is a hygienic procedure, performed at the end of every cycle, for material used for vaccination and medical treatment.	Yes, there is a hygienic procedure, performed at the end of every cycle, for material used for vaccination and medical treatment.
23	Are there any results from analyses, whether official or conducted in self-monitoring, on samples taken from animals or other matrices that	Yes, laboratory analysis results were carefully stored in order to preserve animal health status history.	Yes, laboratory analysis results were carefully stored in order to preserve animal health status history.

	are relevant to human and animal health?		
24	Is there a system for recording health, breeding, and production data for the farm?	A software for data monitoring and visualization was active.	A software for data monitoring and visualization was active.
25	Do stockmen have contact with other pig farms?	No, stockmen worked exclusively for this farm.	No, stockmen worked exclusively for this farm.
26	Is there a visitor log that includes at least the date, name and surname of the visitor, purpose of the visit, and vehicle license plate number?	Yes, visitors were obliged to register with all this information before entering the facility.	Yes, visitors were obliged to register with all this information before entering the facility.
27	Is there documentation confirming the disinfection of the vehicles?	Yes, the driver provides documentation confirming the vehicle cleaning and disinfection.	Yes, the driver provides documentation confirming the vehicle cleaning and disinfection.
28	Does the farm have a designated area for the vehicles cleaning and disinfection located near the entrance of the farm or, in any case, separate from the area designated for animal housing and management?	Yes, the disinfection arch is situated in correspondence with the entry gate to the farm.	Since the farm building corresponds with the farm perimeter and windows are all situated at least 180 cm above the ground, no disinfection arch is required.
29	Are there permanent pressure equipment for the cleaning, washing, and disinfection of incoming vehicles?	Yes, the equipment is permanent.	Not relevant.
30	Are products proven effective against vesicular diseases in pigs and ASF (African Swine Fever) available and used for vehicle disinfection?	Products used for cleaning and disinfection are registered as virucidal, i.e. that are effective against ASF virus.	Not relevant.
31	Is the loading/unloading of live pigs conducted outside the animal housing and management area?	Yes, it was conducted outside the clean area.	Yes, it was conducted outside the clean area.

32	Is there a loading/unloading ramp or corridor for live animals, either permanent or mobile?	There was a mobile ramp for pigs movement.	There was a permanent ramp for pigs movement.
33	Is the loading/unloading of live pigs done using a single vehicle or unit?	Yes, vehicles were used exclusively for this loading/unloading.	Yes, vehicles were used exclusively for this loading/unloading.
34	Is the loading of culled animals conducted outside the animal housing and management area?	Yes, the loading of culled animals is conducted in the dirty area of the farm.	Yes, the loading of culled animals is conducted outside the farm perimeter.
35	Is the loading of culled animals done using a single vehicle or unit?	No, the vehicle is used by other farms as well on the same day.	No, the vehicle is used by other farms as well on the same day.
36	Are the dead animals' carcasses removed from the housing facilities within 24 hours of death, stored in an insulated container or a sealed and functional cold storage cell, located outside the animal management area, and disposed of in accordance with health regulations?	Yes, each morning stockmen are instructed to remove carcasses, so that they are removed within 24 hours. The storage cell is located in the dirty area.	Yes, each morning stockmen are instructed in removing carcasses, so that they are removed within 24 hours. The storage cell is located outside the farm perimeter.
37	Is the loading of dead pigs conducted outside the animal housing and management area?	Yes, the dead pigs loading was conducted in the dirty area.	Yes, the dead pigs loading was conducted outside the farm perimeter.
38	Does the container or cold storage cell where dead animals are stored have a separate access and pathway from the animal housing and management area?	The cold storage cell was located outside of the farm perimeter.	The cold storage cell was located outside of the farm perimeter.
39	Is the area beneath the container or cold storage cell for dead animals suitable for both collecting any leaking materials or liquids and for cleaning and disinfection?	Yes, the cold storage cell was provided with a paved containment basin with raised edges.	Yes, the container was provided with a paved containment basin with raised edges.
40	If piglet carcasses are temporarily stored in the housing facilities while awaiting removal, are the	Yes, the cell could be properly closed, and it keeps the appropriate temperature.	Yes, the cell could be properly closed, and it keeps the appropriate temperature.

	containers used adequately sealed and suitable for their preservation?		
41	Is feed unloading done in a way that prevents contact with other animals?	Yes, the staff in charge were properly trained and aware of biosecurity measures.	Yes, the staff in charge were properly trained and aware of biosecurity measures.
42	Are the feed and/or bedding storage rooms, buildings, or areas covered with grilles or sealed to prevent the entry of other animals, rats, and harmful insects?	Yes, for the feed, that is stored in closed silos. Differently, straw as environmental enrichment is stored in the clean area, but it does not avoid the entry of rats and harmful insects.	Yes, the feed is stored in closed silos and straw as environmental enrichment is stored in a closed deposit.
43	Do the areas beneath the feed silos allow for effective cleaning and drainage of wash water?	Yes, the area beneath the silos is completely paved, allowing proper cleaning and drainage of water.	Yes, the area beneath the silos is completely paved, allowing proper cleaning and drainage of water.
44	Are dairy by-products used for feeding the animals?	No	No
45	If dairy by-products are used for feeding the animals, is there a permit for their use, and is their traceability ensured?	Not relevant.	Not relevant.
46	Is the weighing point used exclusively by the farm?	No, it is the village weighing point, where other vehicles had access.	No, it is the village weighing point, where other vehicles had access.
47	Is swill feeding (the feeding of restaurant waste, cafeteria leftovers, or household food scraps) to animals prohibited?	Yes, it is strictly forbidden, and staff/visitors sign a self-declaration.	Yes, it is strictly forbidden, and staff/visitors sign a self-declaration.
48	Are there separate access points for the transport operations of manure, distinct from those used for the animal housing and management area?	The access points for the transport operations of manure were located out of the farm perimeter.	The access points for the transport operations of manure were located out of the farm perimeter.

49	Are the lands adjacent to the farm used for the spreading of manure from other farms?	No, all surrounding lands were used for manure derived from the farm.	A few adjacent lands were used for manure derived from other farms.
50	Are domestic or pet animals not allowed to access the areas where pigs are housed?	No, they were not.	No, they were not.
51	Are bird nets installed, or is it otherwise ensured that birds cannot enter the buildings?	Net birds are installed, but they are old and not always perfectly functional, meaning that birds can find gaps to enter in the barns.	Since it is a fully sealed barn, birds cannot enter the facility.
52	Do the stockmen and/or visitors engage in hunting or other activities involving contact with wild suids in the 48 hours prior to entering the farm?	No, it is strictly forbidden, and staff/visitors sign a self-declaration.	No, it is strictly forbidden, and staff/visitors sign a self-declaration.
53	Is there a self-declaration from employees of intensive pig farms stating that they do not keep pigs or wild boars raised in a rural setting?	Yes, it is strictly forbidden, and staff/visitors sign a self-declaration.	Yes, it is strictly forbidden, and staff/visitors sign a self-declaration.
54	Is there a ban on introducing feed made from pigs or wild boars from rural sources into the farm?	Yes, visitors/staff signed a self-declaration stating they do not bring in food deriving from suids.	Yes, visitors/staff signed a self-declaration stating they do not bring in food deriving from suids.
55	Is there a ban on feeding pigs with feed made from pigs or wild boars from rural sources?	Yes, staff signed a self-declaration stating they do not feed pigs food derived from suids.	Yes, staff signed a self-declaration stating they do not feed pigs with any kind of food.

Section on Reproduction and Quarantine

56	Are gilts self-produced or introduced from an external genetic site?	They are introduced from an external genetic site	They are introduced from an external genetic site

57	Does the farm have physically and functionally separate facilities for the quarantine of newly introduced breeders?	The quarantine room was exclusive for this porpouse and there was a separate management (air system, feed, equipment), except for manure.	The quarantine room was exclusive for this porpouse and there was a separate management (manure, air system, feed, equipment).
58	Is the all-in/all-out system practiced, along with an appropriate sanitary empty period?	Yes, new gilts arrived only when the quarantine room was completely empty and it was clean and disinfected properly.	Yes, new gilts arrived only when the quarantine room was completely empty and it was clean and disinfected properly.
59	Is the staff dedicated only to animals in quarantine, or is there a specific "clean" area designated for quarantine if staff handle other animals as well?	There was no clean area, but staff were instructed to work in the quarantine as their last job in the working day.	There was no clean area, but staff were instructed to work in the quarantine as their last job in the working day.
60	Do the quarantine facilities have separate pit(s) for waste disposal?	No	Yes
61	Do the quarantine facilities have separate entrance(s)?	Yes, there is a specific entrance only for the quarantine.	Yes, there is a specific entrance only for the quarantine.
62	Are there equipment and facilities available that are dedicated exclusively to quarantine use?	Yes, the equipment and facility were exclusive for quarantine.	Yes, the equipment and facility were exclusive for quarantine.
63	Are there clothing or disposable items (such as coveralls and shoe covers) available that are dedicated exclusively to quarantine use?	Yes, dedicated coveralls and footwear are available for staff/visitors.	Yes, dedicated coveralls and footwear are available for staff/visitors.
64	Is there a planned schedule for diagnostic testing of animals in quarantine?	Yes, when breeders arrived, they were systematically tested for PRRSv.	Yes, when breeders arrived, they were systematically tested for PRRSv.
65	Is documentation available from the source farms that certifies the health status of newly introduced animals?	Yes, documentation regarding breeders' health status was provided at the animals' arrival.	Yes, documentation regarding breeders' health status was provided at the animals' arrival.
66	Is the replacement of breeders carried out at intervals greater than 3 months?	Breeders' replacement was carried out 4 times during 2023, at 3 months intervals.	Breeders' replacement was carried out 4 times during 2023, at 3 months intervals.
67	Is the ultrasound examination performed by external operators?	No, it is performed by farm personnel.	No, it is performed by farm personnel.

68	If artificial insemination is practiced, does the semen come from authorized semen collection centers?	Yes, semen was bought from authorized centers.	Yes, semen was bought from authorized centers.
69	If natural mating is practiced, have the boars undergone the required diagnostic tests for male breeders of the swine species?	Natural mating is not practiced.	Natural mating is not practiced.
70	Are the piglets in the farrowing area intended for more than two farms?	Yes, weaning piglets are destined to 4 different weaning sites.	No, weaning piglets all go to the same site 2.

Checklist Classyfarm Animal Welfare Assessment: Breeding Swine (REV 1/2025)

		Conventional farm	Next-generation farm
1	Number of stockmen.<br><br>The number of staff must be sufficient to ensure the health and welfare of all animals in the operation: check the ratio of staff to animals.	Optimal number of employees (1 employee for 200 or less sows).	Optimal number of employees (1 employee for 200 or less sows).
2	Stockmen's training.<br><br>Training is considered adequate when all staff members have received practical instructions, and at least the owner (or their delegate) has 10 years of experience in the sector or holds a relevant qualification. Additionally, a basic requirement is that either the owner or their delegate, as well as the proprietor, have completed a training course related to pig welfare.	Both the owner and the person responsible have adequate training and more than 10 years of experience in the sector. All staff members have completed a training course on pig welfare.	Both the owner and the person responsible have adequate training and more than 10 years of experience in the sector. All staff members have completed a training course on pig welfare.
3	Number of animal inspections.<br><br>Consider the inspections conducted in the barns with regard to the animals' welfare status.	At least 1 inspection per day.	As the facility was designed and established with a focus on higher animal welfare standards, the staff are specifically trained to pay particular attention to animal welfare. Accordingly, the animals are inspected at least twice daily.

4	Animals' management.<br><br>The pigs are divided into homogeneous groups by sex and/or age and/or category (boars, sows and gilts, piglets, weaners, and fattening pigs).	Pigs were raised in an intensive environment and were divided into homogeneous groups.	Pigs were raised in an intensive environment and were divided into homogeneous groups.
5	Groups management.<br><br>Consider procedures designed to prevent aggressive behavior, such as: forming groups during the evening hours, using odorants that are vaporized, adding feed when entering the pen, providing a greater quantity or different types of manipulable materials, and, in exceptional cases, using tranquilizers on veterinary prescription.	All appropriate measures are implemented to ensure optimal group housing conditions from an animal welfare perspective and to prevent excessive aggression beyond normal social behaviour.	All appropriate measures are implemented to ensure optimal group housing conditions from an animal welfare perspective and to prevent excessive aggression beyond normal social behaviour.
6	Treatment of sick or injured animals.	Injured or sick animals immediately receive proper treatment, and if it does not respond to the given treatment, a veterinarian must be consulted. Injured or sick animals are isolated in a dedicated pen.	Injured or sick animals immediately receive proper treatment, and if it does not respond to the given treatment, a veterinarian must be consulted. Injured or sick animals are isolated in a dedicated pen.
7	Euthanasia of animals: written procedures and training.<br><br>It is necessary to comply with the methodologies outlined in the specific regulations (Regulation (EC) No 1099/2009). Evaluate whether these practices are performed by trained personnel and whether written procedures are in place.	Farm personnel had received proper training and written procedures were in place and available for consultation.	Farm personnel had received proper training and written procedures were in place and available for consultation.
8	Management of feed and daily rations.<br><br>Animals must be provided with adequate food in terms of both quality and quantity: the indicated limits are only an aid for the evaluator, who must still consider all the risk factors of the farm before making a judgment.	Feed and daily rations are appropriate for animal age and category, in collaboration with a nutrition specialist.	Feed and daily rations are appropriate for animal age and category, in collaboration with a nutrition specialist.
9	Feeding frequency.<br><br>Verify the breeder's statements and the situation on the farm: check the feed management schedules in the management system or determine if the feeding is *ad libitum*.	Feeding frequency is considered optimal for all animal categories: gestating sows are fed twice daily; farrowing sows are 4 times per day; piglets have *ad*	Feeding frequency is considered optimal for all animal categories: gestating sows are fed twice daily; farrowing sows are 4 times per day; piglets have *ad*

		libitum access to feed; gilts are fed twice or more per day.	*libitum* access to feed; gilts are have *ad libitum* access to feed.
10	Fiber in the diet of pregnant sows. A sufficient amount is defined as a crude fiber content between 2.23% and 6% of the diet or at least 100 grams per day of straw or hay per sow. These limits serve as a guide for the evaluator, who must still consider all risk factors on the farm before making a judgment.	The diet is formulated by a nutritionist specialized in swine nutrition and meets all the nutritional requirements of the species.	The diet is formulated by a nutritionist specialized in swine nutrition and meets all the nutritional requirements of the species.
11	Energy in the diet of pregnant sows. The diet must include high-energy feed ingredients, such as cereals (verify the energy content per kilogram of feed or according to the daily requirements of the sow).	The diet is formulated by a nutritionist specialized in swine nutrition and meets all the nutritional requirements of the species.	The diet is formulated by a nutritionist specialized in swine nutrition and meets all the nutritional requirements of the species.
12	Mutilations - prohibited procedures. Assess whether mutilation procedures are being performed beyond those exempted by the regulations.	Only operations that cause mutilations permitted by law are performed.	Only operations that cause mutilations permitted by law are performed.
13	Nose ring. The application of nose rings is not permitted; as an exception, it is allowed only in outdoor farming systems.	Nose ring application is not practiced.	Nose ring application is not practiced.
14	Tail docking. Tail docking is prohibited for purposes other than therapeutic ones. As an exception, it is allowed in the presence of tail injuries, subject to verification and subsequent management and environmental improvement, by addressing the enrichment material, provided it is performed by trained personnel (within 7 days of life) or by a veterinarian with anesthesia and prolonged analgesia (beyond 7 days of life).	Tail docking of piglets was performed in compliance with the legislation.	Tail docking was not performed.
15	Castration. Castration of male pigs is prohibited for purposes other than therapeutic ones. As an exception, it is allowed through good surgical practices provided that it is performed on animals intended for specific productions (e.g., DOP, IGP) by trained personnel (within 7 days	Considering that pigs from this scenario were destined to DOP production, male castration is performed according to legislation, using analgesia before 7 days of life.	Considering that pigs from this scenario were destined to DOP production, male castration is performed according to legislation, using analgesia and local anesthesia before 7 days of life.

	of life) or by a veterinarian with anesthesia and prolonged analgesia (beyond 7 days of life and up to sexual maturity).		
16	Grinding or trimming of incisors.<br><br>The incisors reduction is prohibited for purposes other than therapeutic ones. As an exception, it is allowed in the presence of injuries, subject to verification of appropriate management and environmental conditions, provided that it leaves an intact smooth surface and it is performed by a veterinarian or trained personnel within 7 days of life.	Grinding or trimming of incisors are not practiced.	Grinding or trimming of incisors are not practiced.
17	Reduction of boar tusks.<br><br>The boar tusk reduction is prohibited for purposes other than therapeutic ones. As an exception, it is allowed to prevent injuries to other animals or for safety reasons, provided that it is performed by a veterinarian or trained personnel.	Reduction of boar tusks is not practiced.	Reduction of boar tusks is not practiced.
18	Group housing.<br><br>Check the number of sows in production and the number of places in pens (in relation to the farrowing capacity percentage) to assess the structural capacity of the farm regarding the requirement. Verify, on a sample basis, at least 5 sows/gilts in gestation crates the time elapsed since fertilization (using whiteboards or notes on attached forms, computerized records from the farmer, etc.). In case of exceptions for farms with fewer than 10 sows/gilts, check the possibility for the animals to turn around.	The available space and number of places are adequate for the number of sows and gilts housed, ensuring sufficient capacity throughout the production cycle and compliance with animal welfare requirements.	The available space and number of places are adequate for the number of sows and gilts housed, ensuring sufficient capacity throughout the production cycle and compliance with animal welfare requirements.
19	Sows parasite treatment.<br><br>Evaluate the presence/absence of treatments (by reviewing the treatment register or records in Vetinfo or pharmacovigilance) or the presence/absence of evidence of infestations (e.g., visible signs of infestation such as mange dermatitis; reports from the slaughterhouse, e.g., white spots).	Sows’ parasite treatment is routinely performed. If evidence of infestations is detected, individual parasite treatment is performed.	Sows’ parasite treatment is routinely performed. If evidence of infestations is detected, individual parasite treatment is performed.
20	Cleaning of sows in farrowing pens.	When sows were moved to the farrowing pens, they were properly washed, focusing closely on the udders.	When sows were moved to the farrowing pens, they were properly washed, focusing closely on the udders.

	When sows are moved to the farrowing pens, they must be washed or cleaned: assess the procedures declared by the farmer.		
21	Weaning age.	Weaning age was practiced at 35 days of lactation, 2 weeks more than what was permitted by law.	Weaning age was practiced at 35 days of lactation, 2 weeks more than what was permitted by law.
22	Cleaning procedures in facilities specialized for early weaning from 21 to 27 days.<br>In the case of early weaning, piglets must be transferred to facilities that comply with the hygienic and sanitary conditions required by regulations.	Not relevant.	Not relevant.
23	General provisions.<br>Assess whether breeding practices are performed that could harm the 5 freedoms.	No, the 5 freedoms are respected.	No, the 5 freedoms are respected.
24	Lighting for inspection.<br>Verify the availability of inspection lighting, including during nighttime hours.	Lighting is adequate.	Lighting is adequate.
25	Automatic and mechanical equipment - Inspection.	Every automatic or mechanical system (e.g. rations distribution system) was inspected daily in order to verify correct functioning to preserve animal welfare and health.	Every automatic or mechanical system (e.g. rations distribution system) was inspected daily in order to verify correct functioning to preserve animal welfare and health.
26	Ventilation system alarm and backup system.<br>Verify compliance with regulations if the premises ventilation depends on a forced ventilation system.	The ventilation system is a fully forced system and it is provided with a backup system.	The ventilation system is a fully forced system and it is provided with a backup system.
27	Maintenance of animal loading and unloading records (mortality).<br>The loading and unloading registers are present, correctly completed, and kept for the period (3 years) established by current regulations and available for inspection (Legislative Decree No. 200 of October 26, 2010).	The loading and unloading register is present, correctly completed, and kept for the period established.	The loading and unloading register is present, correctly completed, and kept for the period established.

28	Maintenance of pharmacological treatment records.<br><br>The treatment register is present, correctly completed, and kept for the period (5 years) established by current regulations and available for inspection (Legislative Decree No. 193 of April 6, 2006, and Legislative Decree No. 143 of July 24, 2007). Alternatively, computerized recording of treatments on Vetinfo has been adopted, in accordance with Law No. 167 of November 20, 2017.	The pharmacological treatment records are correctly completed on a paper register and also on a computerized system (VetInfo).	The pharmacological treatment records are correctly completed on a paper register and also on a computerized system (VetInfo).
29	Administration of illegal substances.	No illegal substances are administered to animals.	No illegal substances are administered to animals.
30	Presence of shelters in outdoor areas for animals kept outside the buildings.<br><br>Both natural shelters (such as trees, crevices, caves, etc.) and artificial shelters (such as sheds, shelters, etc.) are considered, provided they offer adequate protection in relation to the season and location.	Gestation pens were provided with outdoor areas with shelters for adequate protection.	Since the farm consisted of a single sealed barn, no outdoor area was present.
31	Animals' management.<br><br>The areas used for food preparation/storage are adequately separated and meet the minimum hygiene and sanitary requirements.	Feed preparation and storage areas are adequately separated and meet the minimum hygiene and sanitary requirements, in accordance with Legislative Decree No. 146/2001.	Feed preparation and storage areas are adequately separated and meet the minimum hygiene and sanitary requirements, in accordance with Legislative Decree No. 146/2001.
32	Infirmary.<br><br>In the infirmary, only sick or injured animals should be housed. Check the presence, number, and condition of the infirmary facilities.	An adequate number of infirmaries was present, so that the staff could isolate any animal in need of individual care and treatment.	An adequate number of infirmaries was present, so that the staff could isolate any animal in need of individual care and treatment.
33	Isolation of at-risk or aggressive animals.<br><br>Assess the possibility of temporarily housing animals in an individual enclosure with structural features suitable for the case.	In the facility it was possible to temporarily house animals in an individual enclosure.	In the facility it was possible to temporarily house animals in an individual enclosure.
34	Space in the gestation crate and farrowing pen.	Gestation and farrowing crates comply with the requirements of Legislative	The dimensions exceed the minimum requirements: gestation crates: 1.44 m^2 (65 ×

	The cages must be of a size that allows the animal to rest and stand up without hitting the cage structures.	Decrees No. 122/2011 and 146/2001 and are of sufficient dimensions to allow the animals to lie down and stand up without coming into contact with the crate structures.	222 cm); farrowing pens: 7.2 m², of which 4.48 m² are available to the sow, with 70% solid flooring. Farrowing crates are used for temporary confinement only and are opened 4 days after farrowing, ensuring enhanced freedom of movement and higher welfare conditions.
35	Sows' density in gestation pens.	EU stocking density requirements are complied with: 1.64 m² of total unobstructed floor area per gilt after mating and 2.25 m² per sow when animals are kept in groups, in accordance with Legislative Decree No. 122/2011.	Sows are housed at a density of 2.50 m² per sow in gestation pens, while gilts are provided with 1.80 m² per animal, representing higher welfare conditions than the minimum regulatory requirements.
36	Flooring.<br>Check the type of flooring and the dimensions of the gaps and slats.	The flooring consists of slatted flooring with appropriate slat and gap dimensions, suitable for the category of pigs housed and in compliance with the requirements of Directive 2008/120/EC.	The housing areas are predominantly equipped with solid flooring, except for approximately 30% of the farrowing pen, which consists of slatted flooring compliant with the requirements of Directive 2008/120/EC.
37	Resting area and the possibility to see other animals.	All requirements regarding the resting area are fulfilled. The housing facilities provide comfortable, dry, clean and thermally appropriate resting areas, allowing all animals to lie down simultaneously and to rest and stand up with normal movements. Animals are also able to see other pigs, where applicable.	All requirements regarding the resting area are fulfilled. The housing facilities provide comfortable, dry, clean and thermally appropriate resting areas, allowing all animals to lie down simultaneously and to rest and stand up with normal movements. Animals are also able to see other pigs, where applicable.
38	Absence of harmful materials and equipment for the animals.<br>Enclosures and equipment that animals may come into contact with should not be harmful,	Animals could not come into contact with harmful materials.	Animals could not come into contact with harmful materials.

	have sharp edges, or protrusions that could cause injuries.		
39	Characteristics of flooring and structures.<br><br>The flooring should not be slippery or have rough surfaces and must be suitable for the category of pigs being raised.	The flooring consists of slatted floors appropriate for the category of pigs housed, with a surface that is not slippery or excessively rough, ensuring safe movement and adequate animal welfare, in compliance with the requirements of Directive 2008/120/EC..	The flooring consists entirely of solid floors with straw bedding, appropriate for the category of pigs housed and ensuring safe movement and adequate animal welfare.
40	Temperature, Relative Humidity, and Dustiness.<br><br>Check the type of ventilation present and the microclimatic conditions in the facility, with particular attention to temperature, relative humidity, and dustiness. The indicated limits are merely a guide for the evaluator, who should consider all risk factors of the facility before making a judgment.	Suitable microclimatic conditions are ensured for the animals. The farm is naturally ventilated, except for the farrowing rooms, where controlled ventilation systems with dedicated control mechanisms are in place to maintain appropriate environmental conditions.	Suitable microclimatic conditions are ensured for the animals. The farm is equipped with forced ventilation systems with positive pressure, providing independent ventilation for each room and continuous monitoring of environmental parameters. An alarm system is also in place to promptly detect any malfunction or critical environmental conditions.
41	Presence of harmful gasses.<br><br>The indicated limits must be measured at the time of inspection using appropriate equipment. In the absence of specific equipment, exceptionally, limits (NH3) may be considered exceeded if there is conjunctival redness and tearing in the animals, and similar sensations are experienced by the inspector. The inspector should still consider all risk factors of the facility before making a judgment.	The concentrations of harmful gases are maintained at optimal levels for the animals, with $NH_3 < 10$ ppm and $CO_2 < 3000$ ppm, in accordance with the recommended reference limits.	The concentrations of harmful gases are maintained at optimal levels for the animals, with $NH_3 < 10$ ppm and $CO_2 < 3000$ ppm, in accordance with the recommended reference limits.
42	Noise.<br><br>Typically, under routine conditions, a noise level of 85 dBA can be compared to the noise experienced in heavy traffic.	Animals were not subjected to continuous noises superior to 85 dBA.	Animals were not subjected to continuous noises superior to 85 dBA.

43	Lighting.<br><br>Animals housed indoors should not be kept constantly in the dark or exposed to artificial lighting without an adequate rest period. If the available natural light is insufficient to meet the behavioral and physiological needs of the animals, appropriate artificial lighting must be provided. Typically, under routine conditions, a reference of 40 lux can be considered as a light level that allows for reading a checklist without effort.	A minimum level of artificial lighting of 40 lux is guaranteed for at least 8 hours per day, in compliance with the requirements of Directive 2008/120/EC..	A minimum level of artificial lighting of 40 lux is guaranteed for at least 8 hours per day, in compliance with the requirements of Directive 2008/120/EC.. Windows are also present to provide natural daylight.
44	Feeding: access to feed.<br><br>In the case of controlled feeding, the length of the feeder front for each sow and gilt should be 50 cm to allow simultaneous feeding. In the case of *ad libitum* feeding, it is considered beneficial to have a distribution point per pen, with an optimal distribution point for every 10 animals.	Sows have sufficient space at the trough, while gilts are provided with ad libitum access to feed through a feeding system with one feeding point for every 10 animals, in compliance with the requirements of Directive 2008/120/EC..	Gestating sows are individually fed through an electronic feeding system using individual microchip identification, allowing a tailor-made diet for each sow. Gilts are fed *ad libitum*, with one feeding point for every 10 animals, in compliance with the requirements of Directive 2008/120/EC..
45	Controlled feeding: aggression among sows and gilts in pens.<br><br>The used system ensures that aggression is prevented.	The feeding system ensures that aggressive interactions are avoided.	The feeding system ensures that aggressive interactions are avoided.
46	Feed distributions.<br><br>Evaluate the cleanliness of equipment used for feeding and watering.	Equipment for the provision of feed and water was suitable (free from fecal material and clean).	Equipment for the provision of feed and water was suitable (free from fecal material and clean).
47	Drinking water: availability.<br><br>Check that all pigs have constant access to a continuously functioning source of freshwater. Evaluate the number of waterers present, their accessibility, and their functionality	Presence of functioning, accessible, and clean water troughs, with a minimum of 2 per stall or at least 1 for every 15 animals.	Presence of functioning, accessible, and clean water troughs, with a minimum of 2 per stall or at least 1 for every 15 animals.
48	Materials for exploration and manipulation.<br><br>Verify the presence of materials for exploration and manipulation and ensure that these are available to the animals in all areas.	Chains and wooden blocks are provided as manipulable materials, offering complementary properties and fulfilling the	Daily provision of short, chopped straw as nesting material and environmental enrichment for all sow categories.

		requirements for appropriate enrichment materials.	
49	Free space for natural or assisted farrowing.	Presence of a free area for birthing: a farrowing crate closure that ensures sufficient space beyond the sow's rear).	Presence of a free area for birthing: a farrowing crate closure that ensures sufficient space beyond the sow's rear).
50	Farrowing stall.<br>In the farrowing area, there must be structures to safeguard the piglets and sufficient space for nursing.	In the farrowing area, a dedicated area for piglets is appropriately positioned to protect them, and there is sufficient space for nursing without difficulty.	The area dedicated to piglets is fully enclosed by walls, with an access opening from the pen, ensuring adequate protection, thermal comfort, and sufficient space for nursing without difficulty.
51	Nesting material.<br>Verify the presence of materials.	Paper scraps were used as nesting material.	Straw was available for sows as nesting material.
52	Compliance of flooring for piglets and weaners and heat source.<br>Evaluate the presence of a solid surface for piglets under the sow.	A dedicated solid floor area of 0.60 m^2 is provided for piglets under the sow, together with an adequate heat source.	A dedicated solid floor area of 1.33 m^2 is provided for piglets under the sow, together with a heat source.
53	Space available for boars.	The adult boars have a minimum available floor space of 6 m^2, in compliance with the requirements of Directive 2008/120/EC.	The adult boars have an available floor space of 8.34 m^2, which exceeds the minimum requirement of 6 m^2 established by Directive 2008/120/EC.
54	Contact between boars.<br>Check the dimensions of the boar pens and the possibility of auditory, olfactory, and visual contact.	Possibility of auditory, olfactory, and visual contact.	Possibility of auditory, olfactory, and visual contact.

Figure S1. Economic items (€/productive sow) for conventional (black) and next-generation (grey) scenarios: (A) labor, (B) revenue from cull sows, (C) gilts replacement acquisition, (D) feed, (E) insemination, and (F) medications. The first data point represents the pre-outbreak baseline, calculated from economic data collected during 2022. Subsequent points represent quarterly values annualized to a 12-month equivalent. Panel B represents revenue from culled sow sales and should be interpreted as a revenue rather than a cost item.

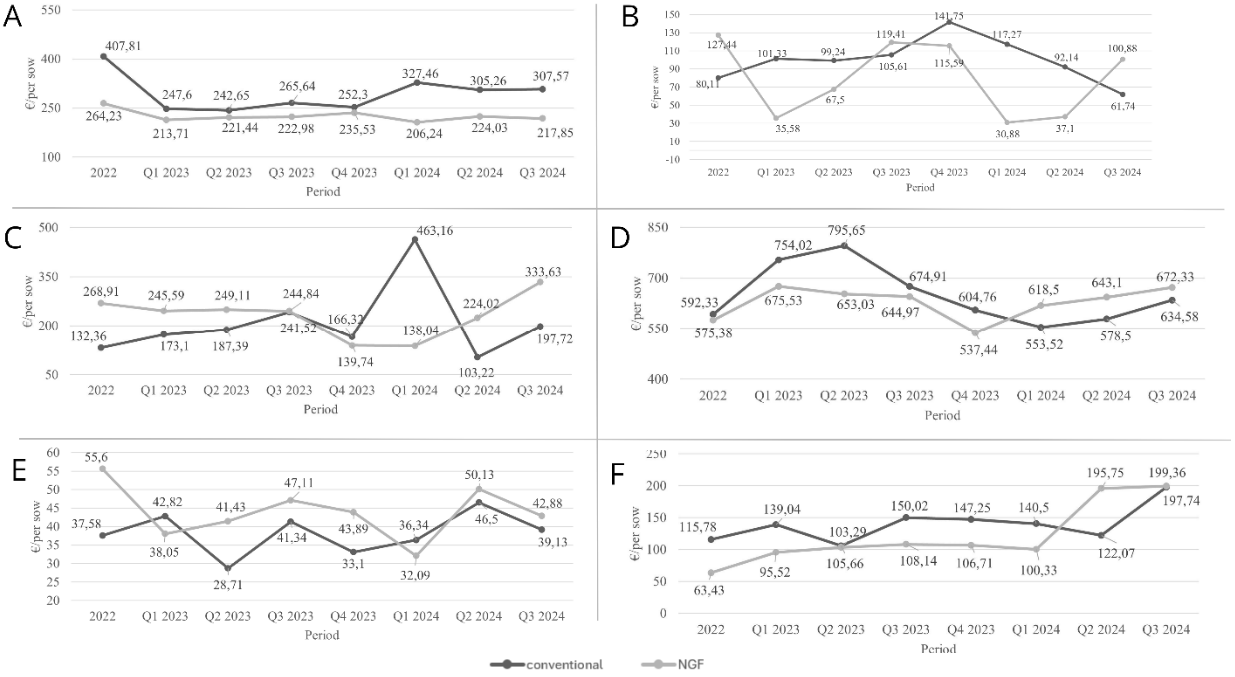

Figure S2. Economic items (€/ weaned piglet) for conventional (black) and next-generation (grey) scenarios: (A) labor, (B) revenue from cull sows, (C) gilts replacement acquisition, (D) feed, (E) insemination, and (F) medications. The first data point represents the pre-outbreak baseline, calculated from economic data collected during 2022. Subsequent points represent quarterly values annualized to a 12-month equivalent. Panel B represents revenue from culled sow sales and should be interpreted as a revenue rather than a cost item.

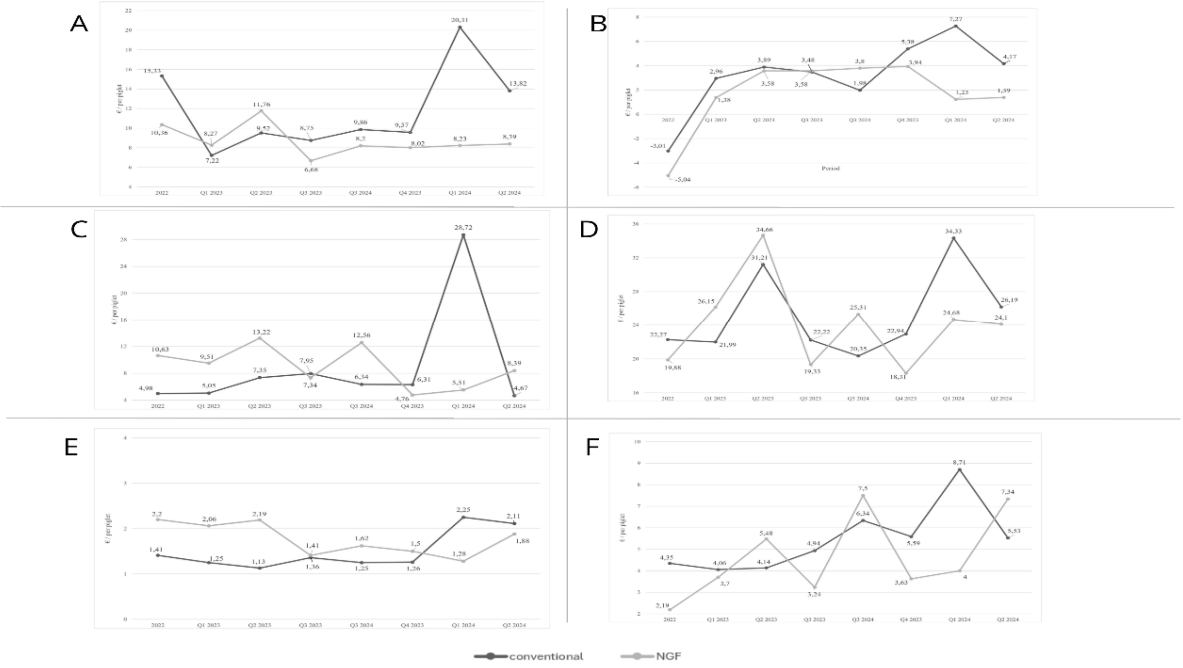

Supplement: Supplementary file 1 [file animals-16-03037-s001.zip › animals-4561090-supplementary.pdf]
